# Stabilizing the Catalyst Layer for Durable and High Performance Alkaline Membrane Fuel Cells and Water Electrolyzers

**DOI:** 10.1021/acscentsci.3c01490

**Published:** 2024-02-15

**Authors:** Chuan Hu, Hyun Woo Kang, Seung Won Jung, Xiaohua Zhang, Young Jun Lee, Na Yoon Kang, Chi Hoon Park, Young Moo Lee

**Affiliations:** † Department of Energy Engineering, College of Engineering, Hanyang University, Seoul 04763, Republic of Korea; ‡ Department of Energy Engineering, Future Convergence Technology Research Institute, Gyeongsang National University, Jinju 52725, Republic of Korea

## Abstract

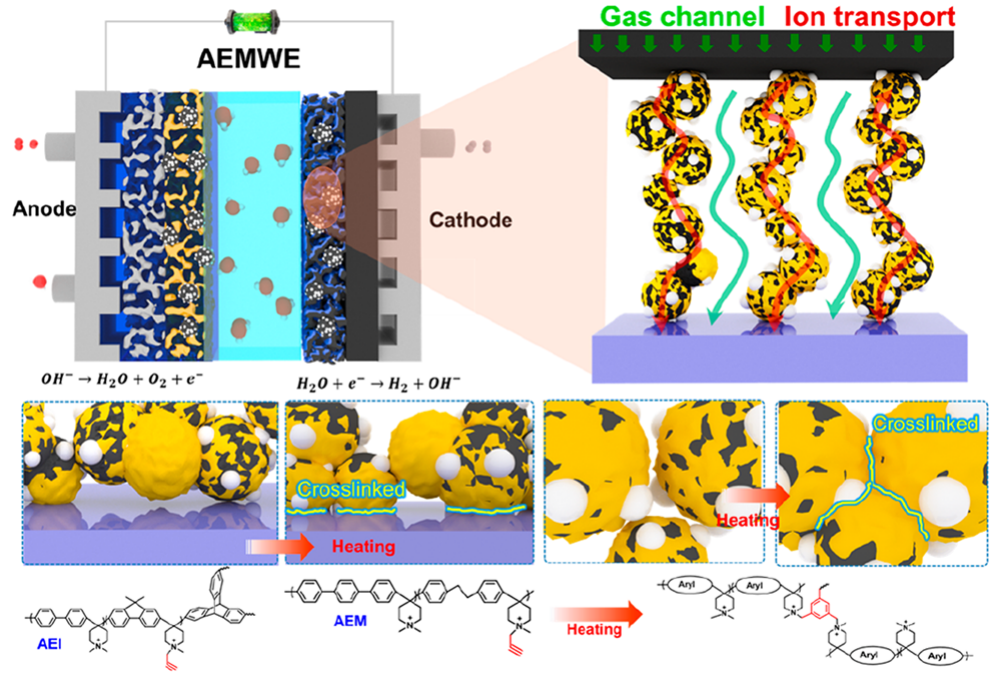

Anion exchange membrane (AEM) fuel cells (AEMFCs) and water electrolyzers (AEMWEs) suffer from insufficient performance and durability compared with commercialized energy conversion systems. Great efforts have been devoted to designing high-quality AEMs and catalysts. However, the significance of the stability of the catalyst layer has been largely disregarded. Here, an in situ cross-linking strategy was developed to promote the interactions within the catalyst layer and the interactions between catalyst layer and AEM. The adhesion strength of the catalyst layer after cross-linking was improved 7 times compared with the uncross-linked catalyst layer due to the formation of covalent bonds between the catalyst layer and AEM. The AEMFC can be operated under 0.6 A cm^–2^ for 1000 h with a voltage decay rate of 20 μV h^–1^. The related AEMWE achieved an unprecedented current density of 15.17 A cm^–2^ at 2.0 V and was operated at 0.5, 1.0, and 1.5 A cm^–2^ for 1000 h.

## Abstract 2

An in situ cross-linking strategy is applied to promote the interactions between the catalyst layer and anion exchange membranes for durable anion exchange membrane fuel cells and water electrolyzers.

## Introduction

Anion exchange membrane fuel cells (AEMFCs) and water electrolyzers (AEMWEs) are promising successors to costly proton exchange membrane fuel cells (PEMFCs) and water electrolysis (PEMWE). These systems have experienced remarkable improvements in the past few years due to the development of highly active nonplatinum group metal (PGM) catalysts, durable and conductive anion exchange membranes (AEMs), and ionomers.^1−5^ The state-of-the-art AEMs achieved hydroxide conductivities greater than 150 mS cm^–1^ at 80 °C with remarkable chemical stability (<10% degradation) in a harsh alkaline environment (80 °C, 1 M NaOH or KOH solution for more than 1000 h).^6−9^ Impressively, state-of-the-art AEMFCs obtained an admirable peak power density of over 3 W cm^–2^,^10^ which pursued a parallel track to commercial PEMFCs. However, the majority of AEMFCs suffer insufficient in situ durability. It is rare for AEMFCs to be stably operated under 0.6 A cm^–2^ for more than 500 h.

The low ionic conductivity of AEMs was thought to be one of the reasons for the poor durability of AEMFCs. Dekel and co-workers pointed out that the improved hydroxide conductivity of AEM enhanced water diffusion through the membrane, which is critical for long-term operation of AEMFCs.^11^ In addition, inappropriate operation conditions also cause the limited fuel cell performance and durability of AMEFCs.^12−14^ As has been well elucidated, the water environment of AEMFCs is much more complicated than that of PEMFCs,^12,13,15^ and the severe water imbalance caused by the water generation reaction in the anode and the water consumption reaction in the cathode can accelerate the degradation of fuel cells.^16^ Moreover, the transport of OH^–^ ions from the cathode to the anode accompanied by 8 water molecules further exacerbated the imbalance of water distributions.^12^ Anode flooding increased the mass transfer resistance of hydrogen, causing voltage loss, while cathode dry-out increased the nucleophilicity of hydroxide ions to attack the functional groups, resulting in degradation of ionomers and an increase in ohmic resistance. Kim, Mustain, and co-workers revealed that the water environment in fuel cells can be optimized by controlling the relative humidity (RH), gas flow rate, and polarity of ionomers in the cathode and anode.^13−15,17−19^

**Figure 1:**
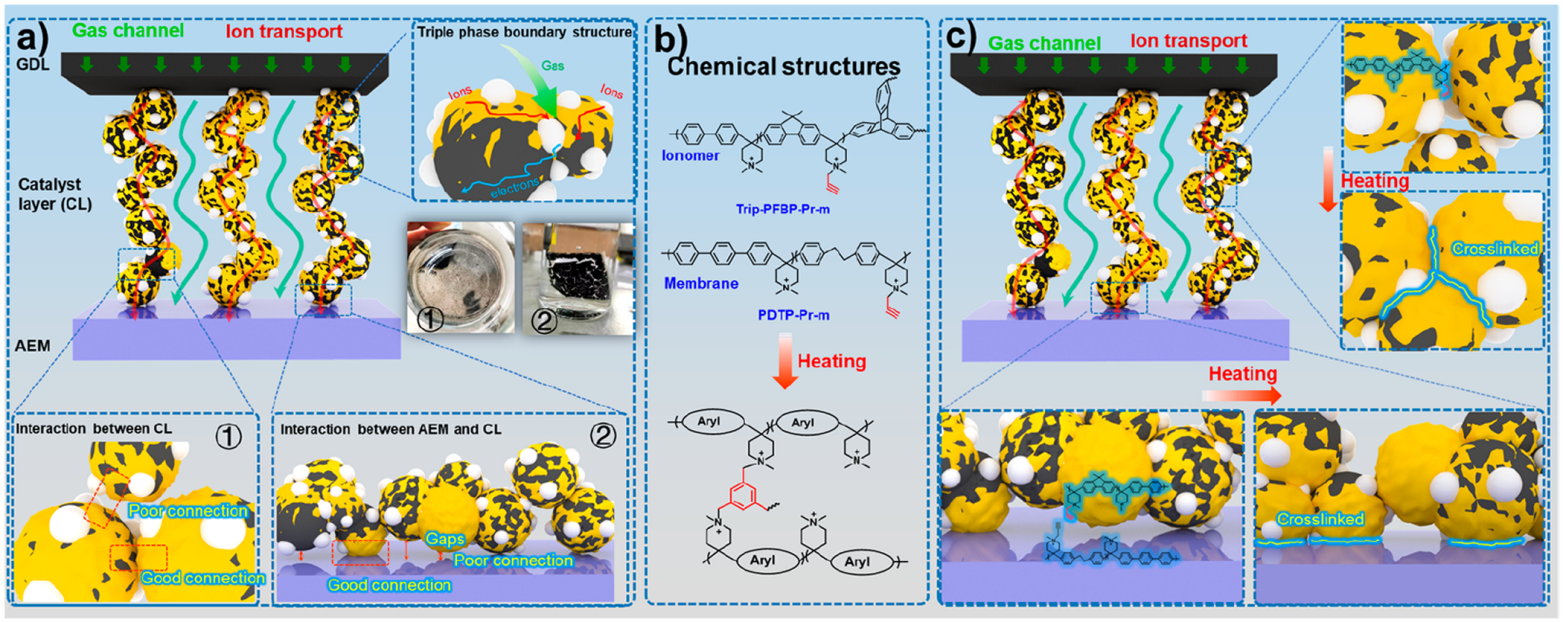
Diagram of the interactions of ionomers in the catalyst layer and the chemical structures of propargyl-grafted poly(aryl-*co*-aryl piperidinium) before and after cross-linking. a) Diagram and digital pictures of the catalyst layer without cross-linking structures. b) The chemical structures of the poly(aryl-*co*-aryl piperidinium)-based AEMs and ionomers before and after cross-linking. c) Diagram of the catalyst layer after cross-linking.

In addition, an unstable catalyst layer is responsible for insufficient fuel cell durability. The catalyst layer is the core component of the fuel cell and is the site of electrochemical reactions that determine the performance and lifespan of AEMFCs. At high-current-density conditions, the rapid water absorption and desorption of the catalyst layer impair the stability of the catalyst layer, resulting in aggregation and detachment of catalyst particles.^20^ Moreover, excessive swelling of ionomers blocks the transport of gases. The ideal catalyst layer should have a loose structure, stable triple-phase boundary structure, uniformly distributed catalyst, durable chemical structure, and good adhesion with the ionomer to decrease the interfacial resistance between the catalyst layer and AEM as well as to improve the stability of the catalyst layer. Very recently, Xu and co-workers applied an ionomer cross-linking strategy for durable AEMFCs by immobilizing catalyst particles in the catalyst layer to promote gas permeability and stabilize the Pt/C nanoparticles.^21^ Similarly, Yan et al. utilized a UV-induced cross-linking strategy to increase the porosity of the catalyst layer and to decrease the mass transport resistance of the catalyst layer.^22^ However, weak interactions between AEM and the catalyst layer also greatly impair the durability of AEMFCs, though it is rarely reported to date. Specifically, the difference in swelling ratio between AEM and the catalyst layer will cause the collapse of the catalyst structure, and even the detachment of the catalyst layer (see Figure 1a). AEMWEs suffer a similar predicament due to bubble generation during the oxygen evolution reaction (OER) in the anode, which will crash the catalyst, causing the dispersion or detachment of the catalyst layer, especially at a high current density.

Here, we propose a novel strategy to stabilize the catalyst layer for durable and high-performing AEMFC and AEMWE by enhancing the interactions between the catalyst layer and AEM by simple heat treatment of the membrane electrode assembly (MEA). Propargyl bromide was used as a cross-linking reagent and was grafted into the backbones of triptycene branched poly(fluorenyl-*co*-biphenyl *N*-methylpiperidine) (Trip-PFBM) and poly(dibenzyl-*co*-terphenyl *N*-methylpiperidine) (PDTM), which were used as the ionomer and AEM, respectively. After heat treatment, the interactions between the catalyst layer and AEM, as well as the catalyst layer itself, were greatly improved due to the cross-linking structure and enhanced the stability of MEA (see Figure 1b–c). In this work, the stability of the catalyst layer was evaluated by measuring the adhesion strength of MEAs. The electrochemical and physical stability of ionomers was systematically investigated and discussed as well. The catalyst layer stabilization strategy is expected to improve the durability of AEMFC and AEMWE and inspire rational MEA design for next-generation AEMFCs and AEMWEs in the future.

## Results and Discussion

Before synthesizing propargyl-grafted polymers, Trip-PFBM and PDTM were prepared as the polymer backbone based on our previous reports.^6,7,23^ The grafting degree of the polymers was controlled by adjusting the feed ratio of propargyl bromide (10%, 30%, and 50%, molar ratio to the piperidinium group) as shown in Figure S1. For convenience, the propargyl-grafted Trip-PFBM and PDTM were denoted as Trip-PFBP-Pr-m and PDTP-Pr-m (m = 10, 30, 50), respectively. After thermal treatment, the cross-linked polymers were referred to as x-Trip-PFBP-Pr-m and x-PDTP-Pr-m.

The chemical structures of Trip-PFBP-Pr-m and PDTP-Pr-m copolymers were characterized by ^1^H nuclear magnetic resonance (^1^H NMR) using DMSO-*d*_6_ as the solvent. As shown in Figure S2, the peak at 4.5 ppm is associated with the methylene protons from the propargyl group, which indicates successful grafting. The grafting degree was calculated by the integral ratio between hydrogen atoms in methylene at 4.5 ppm and the hydrogen atoms in the aromatic ring at 7.0–7.8 ppm.

**Figure 2:**
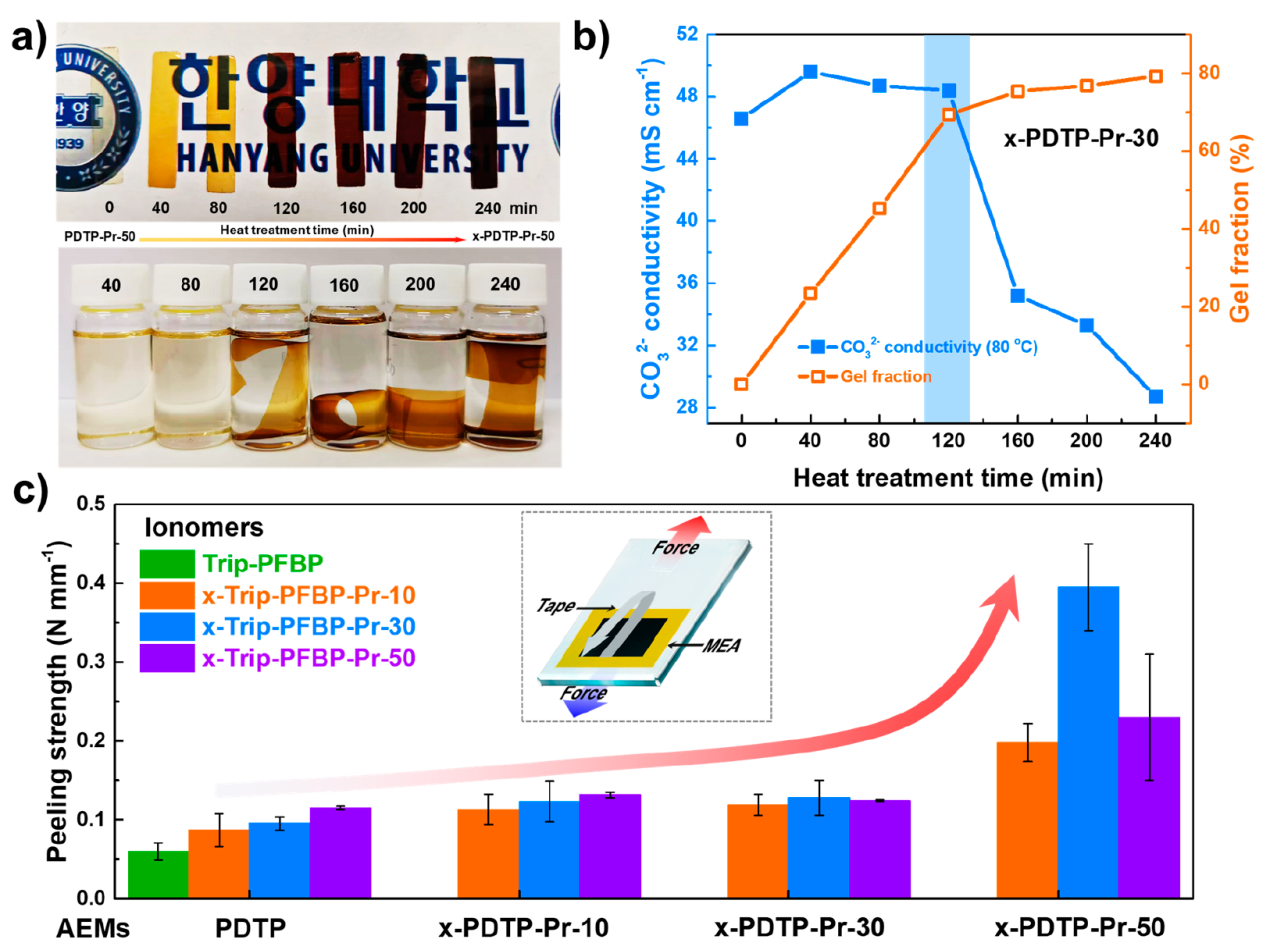
Gel fraction of cross-linked membranes and measurement of the catalyst layer stability. a) The digital pictures of the PDTP-Pr-50 membrane with different thermal treatment times before and after immersion in DMSO solution. b) The gel fraction of membranes with different thermal treatment times. c) The peeling strength measurement of different MEAs at ambient conditions using pristine PDTP and x-PDTP-Pr-m membrane-based AEMs and pristine Trip-PFBP and x-Trip-PFBP-Pr-m based ionomers.

The cross-linking reaction of the propargyl group was triggered after heating at 170 °C under vacuum in a dark environment. To evaluate the degree of cross-linking with heat treatment time, Trip-PFBP-Pr-m and PDTP-Pr-m copolymers were heated from 0 to 240 min. The color of the membrane changed from light yellow to deep brown as heat treatment time increased (see Figure 2a). The gel fractions of polymers were measured by immersing the samples in DMSO solution at 80 °C for 12 h. The samples treated at 170 °C for 40 min showed a low gel fraction and could be dissolved in DMSO solution, while the sample treated for 240 min kept its shape, suggesting a high gel fraction (see Figure 2a and Figure S3). Figure S4 summarizes the positive linear relationship between heat treatment time and gel fraction of membranes.

Fourier-transform infrared spectroscopy (FT-IR) was applied to verify the cross-linking process. The absorption peak at a wavenumber of 2120 cm^–1^ is associated with the alkyne stretching vibration (see Figure S5a).^24^ As heat treatment time increased, the strength of the alkyne absorption peak decreased, suggesting consumption of the propargyl group during the heating process. Figure S5b summarizes the remaining propargyl groups with different heat treatment times by calculating the integral area of the absorption peak of the propargyl group. When the heat treatment time was less than 80 min, the majority of the propargyl group remained unreacted. After 120 min of treatment, more than 70% of propargyl groups reacted, indicating successful cross-linking. The limited heat treatment time should be longer than 120 min. Therefore, the following works are based on heat treatment from 120 to 240 min.

Thermal properties of as-prepared polymers before and after cross-linking were evaluated from 50 to 600 °C under a N_2_ atmosphere. As displayed in Figure S6, the cross-linked polymers possess better thermal stability than pristine polymers.

The water uptake (WU, %) and swelling ratio (SR, %) of prepared membranes in OH^–^ form were evaluated at 30 °C. Trip-PFBP-Pr-m membranes possessed much higher WU and SR than PDTP-Pr-m-based membranes due to their higher ionic exchange capacity (IEC) (see Table 1). As expected, the SR and WU decreased with grafting degree and heat treatment time (see Figure S7a and Figure S7b). For the x-Trip-PFBP-Pr-50 membrane, the WU and SR decreased by half after heat treatment for 240 min. Ionomers with low SR are supposed to achieve higher catalyst layer stability. Similarly, the SR and WU of PDTP-Pr-m membranes decreased with increasing heat treatment time.

**Table 1:** IECs and Physical Properties of Cross-linked AEMs

		WU^c^ (%)		SR^d^ (%)		
Membranes	IEC^a^ (mmol g^–1^)	30 °C	80 °C	30 °C	80 °C	σ^b^ (mS cm^–1^)
PDTP	2.88	117	213	35.4	53.1	168
x-PDTP-Pr-10	2.87	37.8	52.3	15	18.2	149.5
x-PDTP-Pr-30	2.83	31.9	39.5	12.5	16.8	87.8
x-PDTP-Pr-50	2.79	15	23.5	11.5	15	60.6
Trip-PFBP	3.48	530	>1500	86.4	183	NA^e^
x-Trip-PFBP-Pr-10	3.47	356	1240	72.5	170	NA^e^
x-Trip-PFBP-Pr-30	3.42	137	325	35	59.2	97
x-Trip-PFBP-Pr-50	3.37	129	267	30	54.6	82.2

a IEC: ionic exchange capacity, calculated by NMR.

b σ: OH^–^ conductivity at 80 °C.

c WU: water uptake in OH^–^ form.

d SR: swelling ratio in OH^–^ form.

e NA: not available.

To minimize the CO_2_ effect in air during the sample assembling process, the ionic conductivities of the prepared membranes in CO_3_^2–^ form were measured at 80 °C in liquid water. As shown in Figure S8, x-Trip-PFBP-Pr-m and x-PDTP-Pr-m (m = 10, 30) membranes treated at 170 °C for 120 min achieved the highest ionic conductivity. Figure 2b summarizes the CO_3_^2–^ conductivity and gel fraction of the x-PDTP-Pr-30 membrane at different heat treatment times. The conductivity increased up to a cross-linking time of 40–120 min and then decreased dramatically at heat treatment times longer than 160 min. An optimized heat treatment time of 120 min was chosen due to its suitable gel fraction and conductivity. The hydroxide conductivities and Ohmic resistance of x-PDTP-Pr-m and x-Trip-PFBP-Pr-m membranes after 120 min of thermal treatment were measured, as shown in Figures S9 and S10. The x-PDTP-Pr-10 AEM reached the highest OH^–^ conductivity of 149.5 mS cm^–1^ at 80 °C. Among the PFBP series, the x-PFBP-Pr-30 AEM demonstrated a hydroxide conductivity of 97 mS cm^–1^ at 80 °C. The Trip-PFBP-Pr-10 AEM in OH^–^ form has a high water uptake and swelling ratio, which is not suitable for the conductivity measurement. The conductivity of the AEMs was decreased with increasing degree of cross-linking.

The polarity of the polymers was evaluated using contact angle measurements. x-PDTP-Pr-m polymers were used as AEMs, and the contact angle was measured using a membrane shape. While the x-Trip-PFBP-Pr-m polymers acted as ionomers, the contact angles were conducted on MEA shapes. As displayed in Figure S11, the contact angles of the samples treated at 170 °C for 120 min naturally increased with the degree of cross-linking because the hydrophobic cross-linking structure limits the water absorption and membrane swelling. Specifically, the contact angles of x-PDTP-Pr-10, 30, and 50 reached 77.1°, 79.7°, and 91.2°, respectively, which were much higher than that of the benchmark PDTP (59.1°). Impressively, the contact angle of x-Trip-PFBP-Pr-50 reached 74.9°, which is ∼20 times higher than that of benchmark Trip-PFBP (3.6°). The improved hydrophobicity of ionomer and the low swelling upon cross-linking are supposed to promote the contact angle and dimensional stability of the catalyst layer as well as the durability of fuel cells.

As mentioned previously, the weak physical and chemical interactions between ionomers cause the dispersion of catalyst particles when MEA is treated with NaOH solution and water. Meanwhile, the weak connection between the catalyst layer and AEM results in the detachment of the catalyst layer, as displayed in the inset pictures in Figure 1a. To evaluate the interactions with the catalyst layer, the peeling strength of the catalyst layer was measured using a peel-off test. As illustrated in Figure 2c, the 180° peel-off strength was recorded on a universal testing machine (UTM) in ambient conditions. Pristine Trip-PFBP and PDTP were used to prepare benchmark MEAs. The catalyst layer has a thickness of 6.6 μm (see Figure S12). x-Trip-PFBP-Pr-m ionomer-based MEAs achieved much higher peeling strength than that of PDTP&Trip-PFBP (AEM&Ionomer)-based MEAs (see Figure 2c). Specifically, PDTP&x-Trip-PFBP-Pr-50-based MEA possessed a peeling strength of 0.115 N mm^–1^, which is about two times that of the PDTP&Trip-PFBP-based MEA. The peeling strength of MEAs increased again after replacing the pristine PDTP AEM with cross-linked x-PDTP-Pr-m AEMs. Impressively, x-PDTP-Pr-50&x-Trip-PFBP-Pr-30-based MEA reached a peeling strength of 0.395 N mm^–1^, which is ∼7 times higher than that of benchmark MEA. The higher peeling strength of MEA suggests stronger interactions between catalyst layers. The effect of operation temperature and humidity on the peeling strength was further investigated, as shown in Figure S13. As the temperature increased to 80 °C, the peeling strength of x-PDTP-Pr-50&x-Trip-PFBP-Pr-30-based MEA decreased from 0.395 to 0.226 N mm^–1^. In humidified conditions, the peeling strength further decreased to 0.018 N mm^–1^, suggesting that the interaction between the catalyst layer and membrane is vulnerable at high temperatures and wet conditions.

To investigate the enhancement of the interactions between the catalyst layer and AEM (as well as the catalyst layer itself) at the molecular level, ionomer and AEM atomistic models (see Figure S14) were built, and their interaction energies and elongation behavior were observed via Molecular Dynamics (MD) simulations. The simulated models were selected from experimental data with high peeling strengths (ionomers: Trip-PFBP-Pr-30&50, AEM: PDTP-Pr-50). As the cross-linking reaction proceeds, the portion of the cross-linked sites in the simulation models increased, as shown in Figure S15a. Since the Trip-PFBP-Pr-50 model has more cross-linkable functional groups, the portion of the cross-linked sites was much higher than in the Trip-PFBP-Pr-30 model at the same conversion rate. Accordingly, the Trip-PFBP-Pr-50 model with a conversion rate of 41.9% had a much higher number of cross-linked sites compared to Trip-PFBP-Pr-30, which has the same conversion rate. Figure S15b shows the interaction energies of the non-cross-linked and cross-linked models, in which the contribution of the valence bonding energies was calculated to give an insight into the cross-linking reaction in our models. Since the cross-linking reaction forms covalent bonds between two layers, the energy of the valence bonding interaction increases as the conversion rate increases. Consequently, the MD simulation results (also see Figure S16) theoretically confirm the strong interactions based on the covalent bond after cross-linking between the catalyst layer and AEM layer and between the catalyst layers themselves, which can explain the experimental peel-off test results.

To further analyze the stability of the catalyst layer, the ionomer leaching from MEA was monitored by a UV–vis spectrophotometer, as illustrated in Figure S17a. The prepared MEA was treated with NaOH solution and then immersed in H_2_O at 80 °C with N_2_ purging. Due to the swelling of ionomers and weak interactions between catalyst layers, the catalyst layer will detach from the MEA and can be detected by UV–vis. As shown in Figure S17b, PDTP&Trip-PFBP-based MEA shows an obvious absorption peak at a wavelength of 300 nm, which is associated with the absorption of aromatic polymer.^25^ Conversely, the intensity of the absorption peak decreased dramatically when the Trip-PFBP ionomer was replaced with x-Trip-PFBP-Pr-m. The intensity of the absorption peak decreased with increasing degree of cross-linking. Importantly, the absorption peak of the ionomer almost disappeared when the cross-linked AEM was applied. Digital images of the MEA before and after ionomer leaching measurements are shown in Table S1.

**Figure 3:**
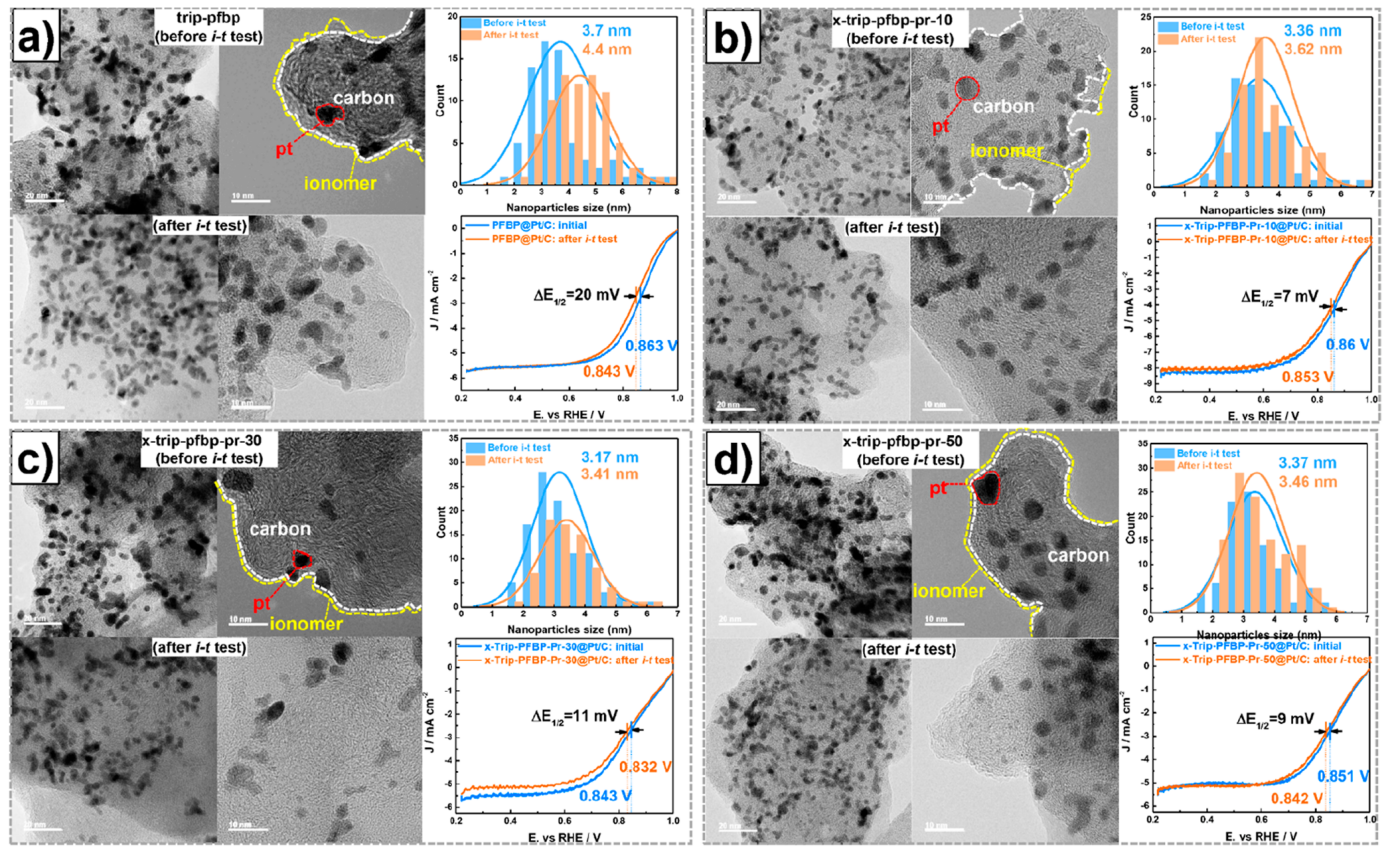
Ex-situ electrochemical stability. TEM images, linear scan voltammograms, and the half-wave potential (hold at −0.5 V vs Ag/AgCl reference) of a) Trip-PFBP, b) x-Trip-PFBP-Pr-10, c) x-Trip-PFBP-Pr-30, and d) x-Trip-PFBP-Pr-50-ionomer-based catalyst ink before and after the *i-t* test. The electrochemical stability of Trip-PFBP- and x-Trip-PFBP-Pr-m-ionomer-based catalyst ink before and after the RDE test in O_2_-saturated aqueous KOH (0.1 M) for 10,000 s.

The electrochemical stability of the catalyst layer was further investigated using the rotating disk electrode (RDE) test. The x-Trip-PFBP-Pr-m ionomers were treated at 170 °C for 120 min and then mixed with Pt/C catalyst to prepare catalyst inks. Trip-PFBP was selected as the benchmark for comparison. After being dispersed in an ultrasonic bath for 1 h, the catalyst inks were deposited onto the surface of RDE to form catalyst layers. The oxygen reduction reaction (ORR) was measured by linear sweep voltammetry (LSV) in an O_2_-saturated aqueous KOH (0.1 M) electrolyte at a rotation speed of 1600 rpm. The stability of catalyst layers was tested at a constant potential of −0.5 V vs Ag/AgCl for 10,000 s. As shown in Figure 3a, the half-wave potential of the Trip-PFBP@Pt/C catalyst decreased from 0.863 to 0.843 V after the durability test. The possible reasons for this are the loss of ionomer as mentioned previously (Figure S17) and the aggregation of catalyst particles. To verify our assumptions, the micromorphology of catalysts was observed using high-resolution transmission electron microscopy (HT-TEM), as displayed in Figure 3a. The Pt particles aggregated into larger sizes after the *i-t* test. Specifically, the average particle size of the Trip-PFBP@Pt/C catalyst increased from 3.7 to 4.4 nm after the short durability test, suggesting its poor stability (Figure 3a). One possible reason for this is the weak interactions between ionomers, which limits them from embedding into the Pt particles, causing agglomeration of catalysts. Conversely, x-Trip-PFBP-Pr-m@Pt/C catalysts showed much more stable ORR LSV performance (Figure 3b), with a half-wave potential degradation less than 11 mV. Aggregation of Pt particles also was greatly inhibited. Specifically, the mean particle sizes of x-Trip-PFBP-Pr-10-, 30-, and 50-ionomer-based catalysts increased from 3.36 to 3.62 nm (Figure 3b), from 3.17 to 3.41 nm (Figure 3c), and from 3.37 to 3.46 nm (Figure 3d), respectively, which were much smaller than that of the benchmark Trip-PFBP-based catalyst (3.7 to 4.4 nm), indicating that the ionomer cross-linking strategy can immobilize the catalyst particles to stabilize energy devices.^21^

**Figure 4:**
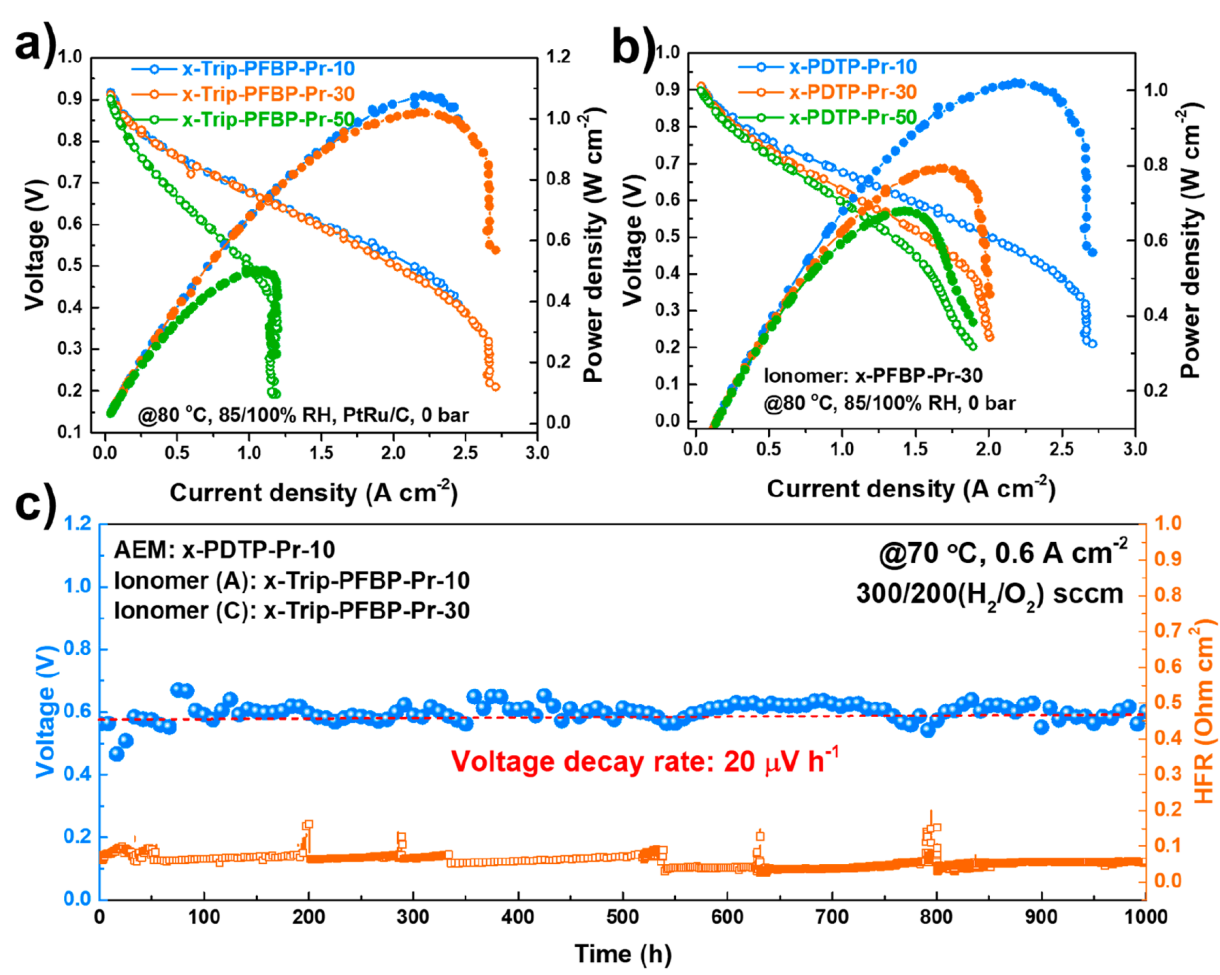
Fuel cell performance and in situ durability. Polarization curves and power density of a) x-PDTP-Pr-10 (25 ± 2 μm) AEM-based AEMFCs with different ionomers and b) x-Trip-PFBP-Pr-30 ionomer-based AEMFC with different AEMs. Test conditions: cell temperature of 80 °C, anode/cathode (A/C) relative humidity (RH) of 85/100%, A/C flow rate of H_2_/O_2_ 1000/1000 mL min^–1^, anode catalyst loading amount of 0.39 mg_PtRu_ cm^–2^, cathode catalyst loading amount of 0.26 mg_Pt_ cm^–2^. c) The in situ durability test of x-PDTP-Pr-10 AEM-based AEMFC at 0.6 A cm^–2^ with x-Trip-PFBP-Pr-10 as the anode ionomer and x-Trip-PFBP-Pr-30 as the cathode ionomer. Test conditions: cell temperature of 70 °C, A/C RH of 94/100%, A/C flow rate of H_2_/O_2_ 300/200 mL min^–1^, anode catalyst loading of 0.4 mg_Pt_ cm^–2^, cathode catalyst loading of 0.4 mg_Pt_ cm^–2^.

The fuel cell performance with different AEM and ionomer combinations and relative humidities (RHs) was evaluated at 80 °C using 1000/1000 mL min^–1^ H_2_/O_2_. Here, 85%/100% (anode/cathode) RH settings were the optimal operation conditions (Figure S18). x-Trip-PFBP-Pr-10 and 30 ionomer-based fuel cells achieved peak power densities (PPDs) greater than 1.0 W cm^–2^ without backpressure, which is twice that of the x-Trip-PFBP-Pr-50 ionomer-based fuel cell (PPD of 0.502 W cm^–2^) (Figure 4a). The improved PPDs were attributed to the high ionic conductivity of x-Trip-PFBP-Pr-10 and 30. Additionally, the x-PDTP-Pr-10 AEM-based fuel cell showed superiority in PPD over x-PDTP-Pr-30 and 50 due to its high WU and conductivity (Figure 4b). Figure S19 compares the power densities of the MEAs (AEM: PDTP-Pr-10; ionomer: Trip-PFBP-Pr-30) before and after cross-linking at 85%/100% RH and 75%/100% RH. Compared with the non-cross-linked MEA, the MEA after cross-linking possesses a higher power density (1.503 W cm^–2^ vs 1.019 W cm^–2^ at 85%/100% RH; 0.909 W cm^–2^ vs 0.735 W cm^–2^ at 75%/100% RH). The improved performance is thought due to the enhanced interaction between the catalyst layer and membrane.

The in situ durability of fuel cells was evaluated under a constant current density of 0.6 A cm^–2^ at 70 °C. Because of the imbalance of water distribution in the water-consuming cathode and water-generating anode sides, an asymmetric ionomer strategy was applied. In other words, different ionomers were used in the cathode and anode sides. However, there is no consensus on the polarity (hydrophilicity or hydrophobicity) of ionomers in each electrode. Figure S20a and Figure S20b show the short-term stability (voltage–time curve) of fuel cells along with high-frequency resistance (HFR) using x-PDTP-Pr-30 as a membrane and x-Trip-PFBP-Pr-30 and x-Trip-PFBP-Pr-10 as ionomers. The combination of hydrophilic x-Trip-PFBP-Pr-10 at the anode and hydrophobic x-PFBP-Pr-30 at the cathode achieved a voltage decay rate of 2.3 mV h^–1^ (Figure S20b), which is half that of a fuel cell with reverse embodiment (voltage decay rate of 5.8 mV h^–1^, Figure S20a). The improved stability is attributed to the increased water gradient, thus promoting the water back diffusion from anode to cathode, consistent with Mustain and Kim’s reports.^10,14^ In addition, the AEM with high conductivity is believed to enhance the longevity of fuel cells.^26^ The x-PDTP-Pr-10 AEM-based fuel cell possesses a much lower voltage decay rate of 0.52 mV h^–1^ than that of the x-PDTP-Pr-30 AEM-based fuel cell (2.3 mV h^–1^) using the asymmetric ionomer strategy (Figure S20c).

Figure 4c shows the 1000 h long-term stability test of the x-PDPT-Pr-10-based fuel cell under the optimal test conditions and asymmetric ionomer strategy (x-Trip-PFBP-Pr-10 at the anode, x-PFBP-Pr-30 at the cathode). During the test, the fuel cell was refreshed by immersing the MEA in an alkaline solution overnight and then washed with deionized water to remove residual alkali. Finally, the refreshed MEA was reassembled in the fuel cell station. Note that we did not replace gas diffusion layers or catalyst layers during the replenishment process, and no catalyst layer detachment from the MEA was observed after the 1000 h test (see the inset picture in Figure 4c). After the replenishment process, the decreased voltage was recovered along with a decreased HFR, which suggests that the decreased voltage during the steady-state operation is probably caused by other reasons (e.g., carbonation or uneven water distribution) and not the chemical degradation of the membrane and ionomer. To the best of our knowledge, AEMFCs that can be operated at 0.6 A cm^–2^ for more than 1000 h have rarely been reported.^10,19^ The excellent in situ stability indicates that the improvement of the interaction between AEM and catalyst layer is an effective way to extend the longevity of AEMFCs and can be a guide and inspiration for future work. Conversely, the non-cross-linked MEA at the same conditions only can be operated for 60 h with a high voltage decay rate of 6274 μV h^–1^, as shown in Figure S21.

**Figure 5:**
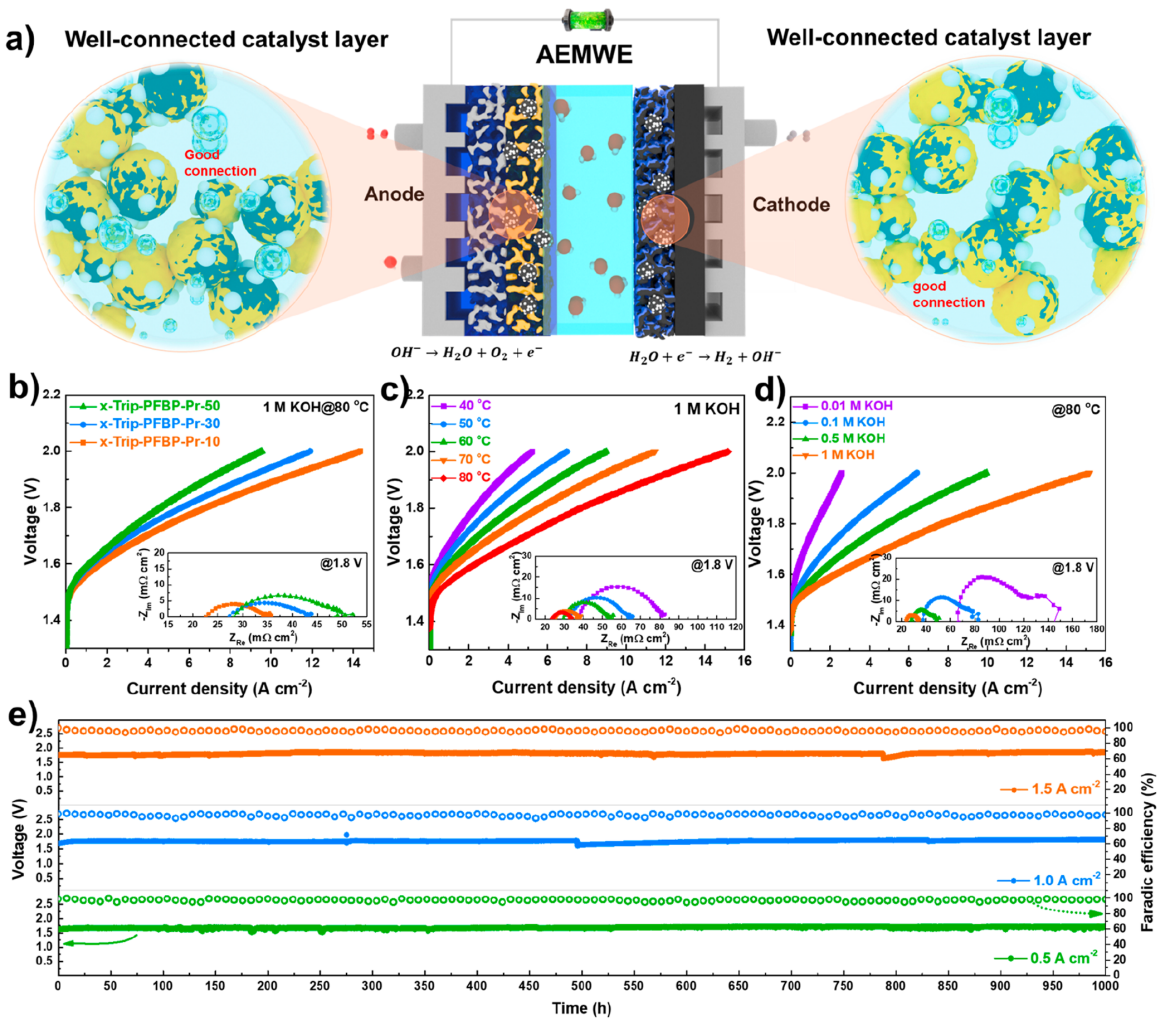
AEMWE performance and in situ durability. a) Diagram of AEMWE and the ionomers at the anode and cathode. b) Linear scan voltammograms (LSV) and potentiostatic electrochemical impedance spectroscopy (PEIS) of an x-PDTP-Pr-10 AEM (20 μm)-based AEMWE at 80 °C in 1 M KOH solution with different ionomers on the two sides. c) The LSV and PEIS of x-PDTP-Pr-10 AEM (20 μm)-based AEMWEs operated at different temperatures (30, 45, 60, and 80 °C) in 1 M KOH solution with an asymmetric ionomer strategy (cathode: x-Trip-PFBP-Pr-10; anode: x-Trip-PFBP-Trip-30). d) The LSV and PEIS of x-PDTP-Pr-10 AEM (20 μm)-based AEMWE operated at 80 °C under different alkaline concentrations (0.01, 0.1, 0.5, and 1 M) with an asymmetric ionomer strategy (cathode: x-Trip-PFBP-Pr-10; anode: x-Trip-PFBP-Trip-30). e) The in situ durability of x-PDTP-Pr-10 AEM (25 μm)-based AEMWE under 60 °C at 1 M KOH solution and different current densities (0.5 A cm^–2^, 1.0 A cm^–2^, and 1.5 A cm^–2^). Test conditions: alkali flow rate of 36 mL min^–1^, anode catalyst of IrO_2_ (2.0 mg cm^–2^), cathode catalyst of PtRu/C (0.7 mg cm^–2^).

In contrast to fuel cells, the electrode reaction of water electrolyzers normally operates in a liquid environment (alkaline solution or water), especially for the anode. The oxygen bubbles generated in the anode will crash the catalyst, causing the dispersion or detachment of the catalyst due to poor connection of catalyst layers, especially at a high current density. Therefore, stabilizing the catalyst layer is crucial for the long-term operation of AEMWEs (see Figure 5a). Figure 5b reveals the WE performance using x-PDTP-Pr-10 (20 ± 2 μm) as the membrane with different ionomers on the two electrode sides.

The PDTP-Pr-10 AEM (20 μm)-based AEMWEs with different ionomers (before cross-linking) were used as reference (see Figure S22). Generally, the non-cross-linked MEAs obtain a higher current density over the cross-linked MEAs (Figure S22a, Figure S22b, and Figure 5b). The promoted current density is associated with their low Ohmic resistance (Figure S22c and Figure S22d). Specifically, the non-cross-linked MEA (AEM: PDTP-Pr-10; ionomer: Trip-PFBP-Pr-10) possesses the highest current density of 15.5 A cm^–2^ at 2 V and 80 °C in a 1 M KOH solution. After thermal treatment, the current density of the MEA (AEM: x-PDTP-Pr-10; ionomer: x-Trip-PFBP-Pr-10) was slightly decreased to 14.34 A cm^–2^ at the same conditions along with an increased R_ohm_ of 22.58 mΩ cm^–2^ and a R_charge_ of 12.28 mΩ cm^–2^. Due to the dried cathode strategy, x-Trip-PFBP-Pr-30 ionomer-based AEMWE shows a lower current density (11.9 A cm^–2^ at 2.0 V) compared with x-Trip-PFBP-Pr-10 ionomer-based AEMWE, which is thought to be the low water absorption capability of the cathode ionomer.^27^ In that case, we applied an asymmetric ionomer strategy for WE performance and durability tests, where x-Trip-PFBP-Pr-10 and x-Trip-PFBP-Pr-30 were used as cathode and anode ionomers, respectively. An improved current density of 15.17 A cm^–2^ at 2.0 V was reached at 80 °C by applying the asymmetric ionomer strategy (Figure 5c). The current density naturally decreased with temperature due to the increased R_ohm_ and R_charge_ (inset figure in Figure 5c). Figure 5d reveals the dependence of WE performance on alkali concentration. AEMWE operating in a concentrated alkaline solution exhibited a higher current density due to the improved conductivity (R_ohm_ of 66 mΩ cm^–2^ in 0.01 M KOH vs R_ohm_ of 23 mΩ cm^–2^ in 1 M KOH) and electrode reaction activity (R_charge_ of 79 mΩ cm^–2^ in 0.01 M KOH vs R_charge_ of 10 mΩ cm^–2^ in 1 M KOH) (inset figure in Figure 5d). Nevertheless, the AEMWE performance in 0.1 M KOH solution was much higher than the majority of reported AEMWEs operated in a 1 M KOH solution.^28−30^

The in situ durability of x-PDTP-Pr-10 (25 μm)-based AEMWE was evaluated at 60 °C in a 1 M KOH solution under different current densities (0.5, 1.0, and 1.5 A cm^–2^) by applying an asymmetric ionomer strategy, as shown in Figure 5e. A dry cathode strategy was applied during the durability test. The AEMWE operated at 0.5 A cm^–2^ displayed a low initial voltage of 1.63 V and a voltage decay rate of 88 μV h^–1^ for 1000 h. As current density increased to 1.0 A cm^–2^, the initial voltage of AEMWE naturally increased to 1.70 V with a maintained low voltage decay rate of 98 μV h^–1^, suggesting its exceptional stability. A current density of 1.5 A cm^–2^ is a quite harsh condition and a challenge for the long-term operation of AEMWE as it generates a large amount of gas bubbles in a short time, resulting in the collapse of the morphology of the catalyst layer. However, the prepared AEMWE with a stabilized catalyst layer can be continuously operated at 1.5 A cm^–2^ for 1000 h with a voltage decay rate of 96 μV h^–1^. More importantly, the AEMWE operated at that harsh condition also exhibited an excellent Faradaic efficiency greater than 95% during the test, indicating the gas tightness of the MEA. Conversely, the non-cross-linked MEA shows a severe voltage increase from 1.65 to 1.96 V during a short operation time at the same conditions (Figure S23). To the best of our knowledge, few AEMWEs can be operated in severe conditions for 1000 h. Our results suggest that stabilization of the catalyst layer is a promising strategy for promoting the performance and stability of AEMWEs.

## Conclusion

In summary, we proposed a facile approach to promote the stability of the catalyst layer by enhancing the interactions between the catalyst layer and AEM through thermal cross-linking. First, we incorporated a thermally cross-linkable propargyl group into the poly(aryl-*co*-aryl piperidinium) backbone. In contrast to the high reactivity of double bonds, the propargyl group has better stability, allowing retention of cross-linkable activity of AEM after being cast as membranes. Subsequently, our experimental and molecular dynamic simulation work revealed the behavior and mechanism of the interaction between the AEM and catalyst layer. As a result, the cross-linked membranes achieved improved dimensional stability and hydrophobicity due to the formation of cross-linked structures. The stabilized catalyst layer displayed ∼7 times higher adhesion strength and better catalyst and ionomer stability than that of the benchmark of un-cross-linked MEA. Importantly, the related AEMFC can be stably operated under 0.6 A cm^–2^ at 70 °C for 1000 h with a low voltage decay rate of 20 μV h^–1^. Moreover, the related AEMWE achieved an unprecedented current density of 15.17 A cm^–2^ at 2.0 V in 1 M KOH solution and can be continuously operated at 0.5, 1.0, and 1.5 A cm^–2^ at 60 °C for 1000 h. This research provided insight into the development of high-performance and durable AEMFCs and AEMWEs.

## Experimental Section

### Synthesis of Propargyl-Grafted Polymers

Before the synthesis of propargyl-grafted polymers, triptycene branched poly(fluorenyl-*co*-biphenyl methylpiperidine) (Trip-PFBM) and poly(dibenzyl-*co*-terphenyl methylpiperidine) (PDTM) were synthesized according to our previous reports.^6,31^ Taking the synthesis of PDTP-Pr-10 as an example, PDTM (43.7 g, 94.2 mmol) was dissolved in 500 mL of DMSO to form a clear solution. Subsequently, potassium carbonate (39 g, 282.2 mmol) and propargyl bromide (1.12 g, 9.42 mmol) were added to the solution and reacted at room temperature for over 24 h. Then, an excess amount of CH_3_I (40 g, 282.2 mmol) was added to the mixture solution and reacted for another 24 h in a dark environment. Finally, the mixture solution was precipitated in 2000 mL of ethyl acetate. The white powder was washed with ethyl acetate and deionized water two times and then dried in a vacuum oven at 45 °C for 24 h. The synthesis of PDTP-Pr-30, PDTP-Pr-50, and Trip-PFBP-Pr-m (m = 10, 30, 50) were performed similarly.

### Cross-Linked Membrane Preparation

Typically, PDTP-Pr-m and Trip-PFBP-Pr-m polymers were dissolved in DMSO to form 5 wt % solutions. After filtering with a PTFE-based filter (pore size 1 mm), the polymer solutions were cast onto a glass plate and dried in a vacuum oven at 80 °C for 24 h. Transparent films were obtained after peeling off from the glass plate. To form cross-linked membranes, the fabricated membranes were heated at 170 °C under vacuum in a dark environment for different times (0–240 min, 40 min increments) to initiate the trimerization reaction or coupling reaction. Finally, cross-linked membranes (x-PDTP-Pr-m and x-Trip-PFBP-Pr-m) were obtained.

### Proton Nuclear Magnetic Resonance

The chemical structures of PDTP-Pr-m and Trip-PFBP-Pr-m polymers were verified via proton nuclear magnetic resonance (^1^H NMR, VNMRS 600 MHz, Varian, CA, USA) using DMSO-*d*_6_ as a solvent.

### Gel Fraction Measurement

The cross-linking reaction of polymers was verified by determining the gel fraction. Briefly, the cross-linked membranes (x-PDTP-Pr-m and x-Trip-PFBP-Pr-m) were cut into a rectangular shape, and the weights of the samples were recorded as *m*_1_. Subsequently, the samples were immersed in a DMSO solution at 80 °C for 24 h. The insoluble solids were collected by filtration and then dried in a vacuum oven at 100 °C for 24 h to remove residual solvent. Finally, the weights of the solids were recorded as *m*_2_. The gel fraction of cross-linked polymers was calculated using the following equation.
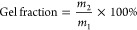
1

### Fourier-Transform Infrared Spectroscopy

Except for gel fraction measurement, Fourier-transform infrared spectroscopy (FT-IR, Nicolet 6700, Thermo Scientific, MA, USA) was used to verify the cross-linking reaction of the propargyl group after being treated at 170 °C for different times. The calibrated area of the characteristic peak was analyzed and calculated using OMNIC 8.0 software.

### Water Uptake and Swelling Ratio

The water uptake (WU, %) and swelling ratio (SR, %) of cross-linked membranes in OH^–^ form was evaluated by measuring the water adsorption and dimensional difference at the dry and wet states, respectively. Typically, the membranes were cut into a rectangular shape, with the dry length recorded as *L*_dry_, then immersed in a 1 M NaOH solution at room temperature for 24 h to exchange ions to OH^–^ form. After washing with deionized (DI) water several times, the membranes were stored in DI water at 30 °C to reach equilibrium. The wet weight and length were recorded as *W*_wet_ and *L*_wet_, respectively. The WU and SR can be calculated according to the following equations.
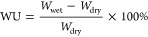
2
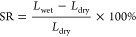
3

### Ionic Conductivity

The CO_3_^2–^ conductivity of the membranes was measured using an impedance analyzer (VSP and VMP3 Booster, Bio-Logic SAS, Grenoble, France). Prior to measurement, the membranes were immersed in 1 M K_2_CO_3_ solution at room temperature for 24 h to exchange ions to CO_3_^2–^ form. Subsequently, the membranes were washed with DI water to remove residual salt. The ohmic resistance of the samples was recorded at 80 °C in liquid water. The CO_3_^2–^ conductivity (σ, mS cm^–1^) of the samples was calculated using the following equation.
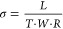
4where *L* (cm) denotes the effective length of the samples between two platinum electrodes. *W* (cm) and *T* (cm) are the width and thickness of the samples, respectively. *R* (kΩ) is the resistance of the samples.

### Thermal Properties

The thermal properties of the samples were evaluated using thermogravimetric analysis (TGA; Q500, New Castle, DE, USA) from 50 to 600 °C under a N_2_ atmosphere. Prior to measurement, the samples were stabilized at 100 °C under N_2_ for 10 min to remove adsorbed water.

### Contact Angle

To evaluate the polarity of the cross-linked membranes and ionomers, static water contact angles of the samples were measured using a contact angle analyzer (SEO Phoenix 300, Suwon, South Korea).

### Peeling Strength Measurement

The adhesive ability of catalyst layers was evaluated by measuring the peeling strength using a UTM (AGS-500NJ, Shimadzu, Tokyo, Japan) at room temperature. Prior to measurement, the Pt/C catalyst combined with ionomers was sprayed onto the surface of membranes. Subsequently, the prepared catalyst-coated membrane (CCM) was treated at 170 °C under vacuum for 2 h to form cross-linking structures. After that, the cross-linked MEA was fixed on a plate with tape attached to the surface of the catalyst layer.

### Ionomer Leaching Measurement

The stability of the catalyst layer was evaluated by measuring the ionomer leaching. The cross-linked CCM was immersed in 1 M NaOH solution at room temperature for 24 h and then immersed in DI water at 80 °C under a N_2_ atmosphere for another 24 h. DI water was used to detect ionomer leaching using a UV–vis spectrophotometer (SPECORD 200, Analytik Jena, Jena, Germany) in the wavelength range from 200 to 600 nm.

### Electrochemical Test of the Catalyst Inks

Electrochemical testing of the catalyst inks was performed using a four-point probe alternating current (AC) impedance analyzer (VSP and VMP3 Booster, Bio-Logic SAS, Grenoble, France) with a three-electrode system and a rotation system (RRDE-3A, ALS, Japan). Prior to measurement, the Trip-PFBP-Pr-m polymers were dissolved in DMSO to form a 5 wt % solution. The ionomer solutions were added into the ampules, which were then sealed with a fire gun. Subsequently, the ampules were heated in an oven for 2 h in a dark environment to form a cross-linked structure. The catalyst inks were prepared by mixing ionomers, Pt/C (Hispec 4000, 40 wt % Pt), DI water, and isopropanol in a 10 mL glass vial, which was then placed in an ultrasonic bath at 0 °C for 1 h. Then, 20 mL of the catalyst ink was dropped onto the glassy carbon rotating disk electrode (GC-RDE, 0.19625 cm^2^) and dried at ambient conditions to obtain a Pt loading of 0.23 mg cm^–2^. Linear sweep voltammetry (LSV) measurements were conducted in an O_2_-saturated aqueous KOH (0.1 M) electrolyte at a rotation speed of 1600 rpm from 0.2 to 1.0 V. The sweep rate was fixed at 10 mV s^–1^. The durability of the catalyst was evaluated at a constant potential of −0.5 V vs Ag/AgCl at the same conditions as in the LSV test.

### Electron Microscopy Analyses

The morphologies of the catalyst inks before and after the electrochemical tests were observed using a transmission electron microscope (TEM, JEM-ARM200, JEOL, Japan) at 200 kV.

### Fuel Cell Performance and Durability Measurement

The polarization and power density curves were measured in a fuel cell station (CNL energy, Gimpo, Gyeonggi-do, Korea). Prior to measurement, the catalyst-coated membranes (CCMs) were prepared using a previously reported method.^32^ Specifically, Trip-PFBP-m (5 wt % in DMSO), Pt/C (HISPEC 4000, 40 wt % Pt), or PtRu/C (HISPEC 10000, 40 wt % Pt, 20 wt % Ru), along with DI water and IPA were mixed and dispersed in an ultrasonic bath for 1 h. Subsequently, the catalyst inks were sprayed onto the surface of the PDTP-Pr-m membranes to prepare CCM. Thereafter, the CCM was treated in a vacuum oven at 170 °C for 2 h in a dark environment to form cross-linking structures. After the CCM was cooled to room temperature, the CCM was immersed in a 1 M KOH solution to exchange ions to OH^–^. Finally, CCM was washed with DI water to remove residual alkali and assembled with two sheets of gas diffusion layers (GDL, SGL 22BB, Sigracet), gaskets, and bipolar plates at a torque of 6.78 N m without hot pressing. During the performance measurement, the fuel cell was activated at a constant voltage of 0.5 V at 70 °C and 100% RH until a constant current was achieved. After that, the cell temperature and gas humidity were changed to their respective set points, and the polarization curves and power density were recorded at a voltage scan rate of 0.01 V s^–1^.

The in situ stability of the fuel cell was measured under a constant current density of 0.6 A cm^–2^ at 70 °C. The MEA preparation for the durability test is different from that for IV curve measurement. Specifically, Pt/C (46.6 wt % Pt, Tanaka, Japan) was used as the electrode catalyst with a loading amount of 0.6 mg cm^–2^ on both sides. To prepare the MEA, the catalyst inks were separated into two parts. Half of the catalyst inks were sprayed onto the surface of the AEMs to prepare CCMs. The rest of the catalyst ink was sprayed onto the surface of the gas diffusion layers (Toray H090) to prepare gas diffusion electrodes (GDEs). After that, the CCMs and GDEs were immersed in 1 M KOH solution to exchange ions and were then combined with gaskets at an assembling force of 6.78 N m^–1^.

### Water Electrolysis Performance and In Situ Durability Measurement

Water electrolysis and in situ durability were assessed using an electrochemical station (VSP, Bio-Logic SAS, Grenoble, France) in combination with a current booster (VMP3 Booster, Bio-Logic SAS, Grenoble, France, with a current operation range of 0 to 80 A). IrO_2_ (Alfa Aesar, MA, USA) and PtRu/C (Hispec 12000, Pt 40%, Ru 20%) were used as anode and cathode catalysts, respectively. The typical MEA manufacturing process was as follows: IrO_2_ (26 mg), ionomer (2.88 mg), DI water (0.5 mL), and IPA (0.5 mL) were mixed in a glass vial and then dispersed in an ultrasonic bath for 1 h for use as an anode catalyst ink. The cathode catalyst ink was prepared similarly using PtRu/C (15.5 mg), ionomer (5.17 mg), DI water (0.1 mL), and IPA (0.9 mL). After that, the well-dispersed catalyst ink was sprayed onto the surface of the membrane, and the CCM was thermally treated in a vacuum oven at 170 °C for 2 h. Finally, the CCM was immersed in 1 M KOH solution at 60 °C to exchange ions to OH^–^. Finally, the CCM was assembled into a single AEMWE cell with a Ni fiber plate (Dioxide Materials, USA), carbon paper (Sigracet 22BB, SGL carbon, Germany), gaskets, and gold-coated nickel plates. The linear sweep voltammetry was measured in alkaline solutions (1, 0.5, 0.1, and 0.01 M KOH) by scanning the voltage from 1.2 to 2.0 V. The in situ durability of AEMWE was evaluated at constant current densities of 0.5, 1.0, and 1.5 A cm^–2^ under 60 °C at 1 M KOH solution for 1000 h. The Faradaic efficiency was simultaneously recorded during the test.

### Simulation Method

We used the Materials Studio package (Dassault Systems, BIOVIA Corp., USA). Molecular dynamics (MD) calculations were conducted using the Forcite module, and 3D amorphous models were constructed using the Amorphous cell module. MD simulations used the Condensed-phase Optimized Molecular Potentials for Atomic Simulation Studies III (COMPASS III) force field.^33−35^ We initially built non-cross-linked 3D models of the Trip-PFBP-Pr-m (m = 30, 50) ionomers and the Trip-PFBP-Pr-50 AEM, separately. To obtain a stable structure, geometric optimization was performed on the layer structure of the ionomer–AEM–ionomer, combining each ionomer model with the AEM model. We performed MD simulations on the ionomer–AEM–ionomer layers at 298 K using the NPT (constant atom number, constant pressure, and temperature) ensemble. The Andersen method was used to control the temperature with fixed constants, and the Berendsen method was used to control the pressure with a damping constant of 1 ps.^36,37^ We calculated MD parameters of electrostatic and van der Waals interactions using the particle–particle mesh (PPM) summation method. Subsequent cross-linking simulations were conducted to determine the interaction energies between ionomer–ionomer and ionomer–AEM pairs, constructing structures based on the layer’s cross-linking degree. Furthermore, we conducted elongation simulations to evaluate the mechanical strength characteristics of the ionomer–AEM–ionomer layers to determine their degree of cross-linking. In the elongation simulations, the layer model was elongated by 0.03% along each cycle’s vertical axis, with the other two axes compressed to preserve volume. The density of the model was equilibrated using the NPT ensemble at 298 K and 1 atm for 10 ps, and this procedure was carried out 60 times.^38^

## Supporting Information Available

Detailed experimental descriptions, ^1^H NMR, solubility, gel fraction, FT-IR, TGA, water uptake, swelling ratio, conductivity, water contact angle, MD simulations, UV absorption, fuel cell performance, fuel cell durability, water electrolysis performance, water electrolysis durability (PDF)
Transparent Peer Review report available (PDF)
