# Supplementary material for: Stabilizing the Catalyst Layer for Durable and High Performance Alkaline Membrane Fuel Cells and Water Electrolyzers
Source: ACS Cent Sci. 2024 Feb 15;10(3):603–14. doi: 10.1021/acscentsci.3c01490 (PMC10979504; doi:10.1021/acscentsci.3c01490)
Supplement: Supplementary file 1 — oc3c01490_si_001.pdf [file oc3c01490_si_001.pdf]

Supporting Information

Stabilizing the catalyst layer for durable and high performance alkaline membrane fuel cells and water electrolyzers

Chuan Hu[1], Hyun Woo Kang[2], Seung Won Jung[1], Xiaohua Zhang[1], Young Jun Lee[1], Na Yoon Kang[1], Chi Hoon Park[2], Young Moo Lee[1]*

[1] Department of Energy Engineering, College of Engineering, Hanyang University, Seoul 04763, Republic of Korea.

[2] Department of Energy Engineering, Future Convergence Technology Research Institute, Gyeongsang National University, Jinju 52725, Republic of Korea

E-mail of corresponding author: ymlee@hanyang.ac.kr

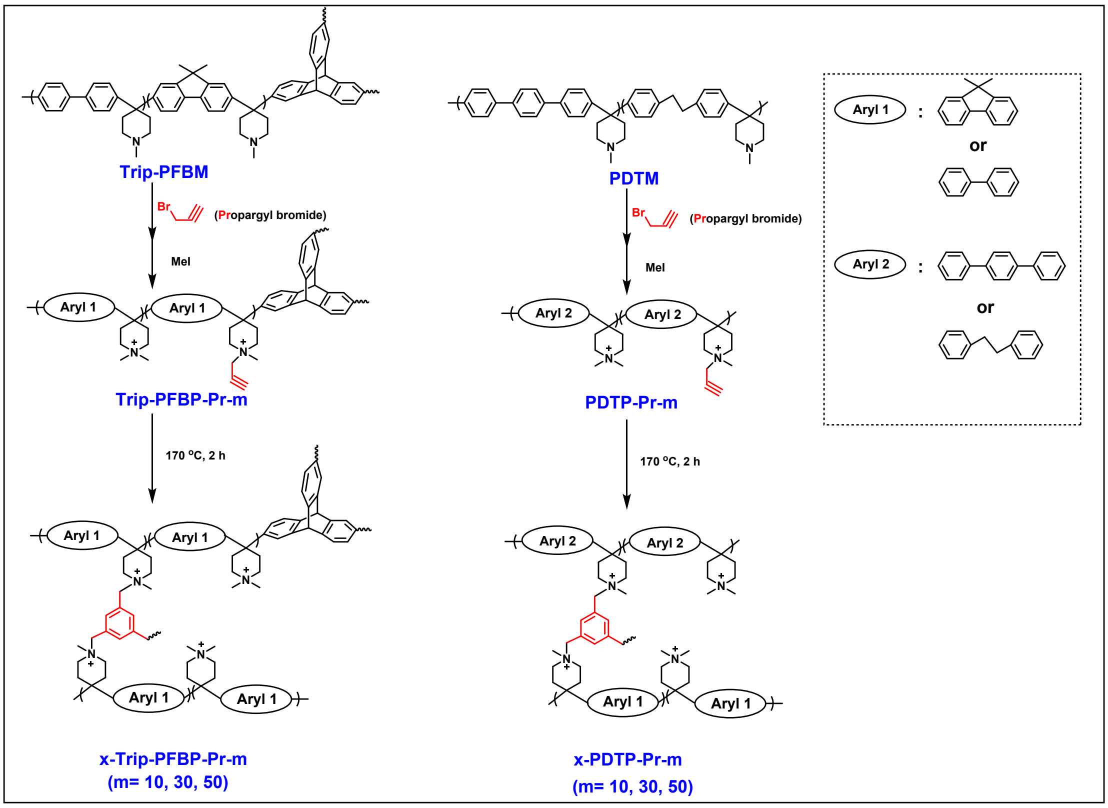

Figure S1. The synthesis of x-Trip-PFBP-m and x-PDTP-Pr-m (m=10, 30, 50) polymers.

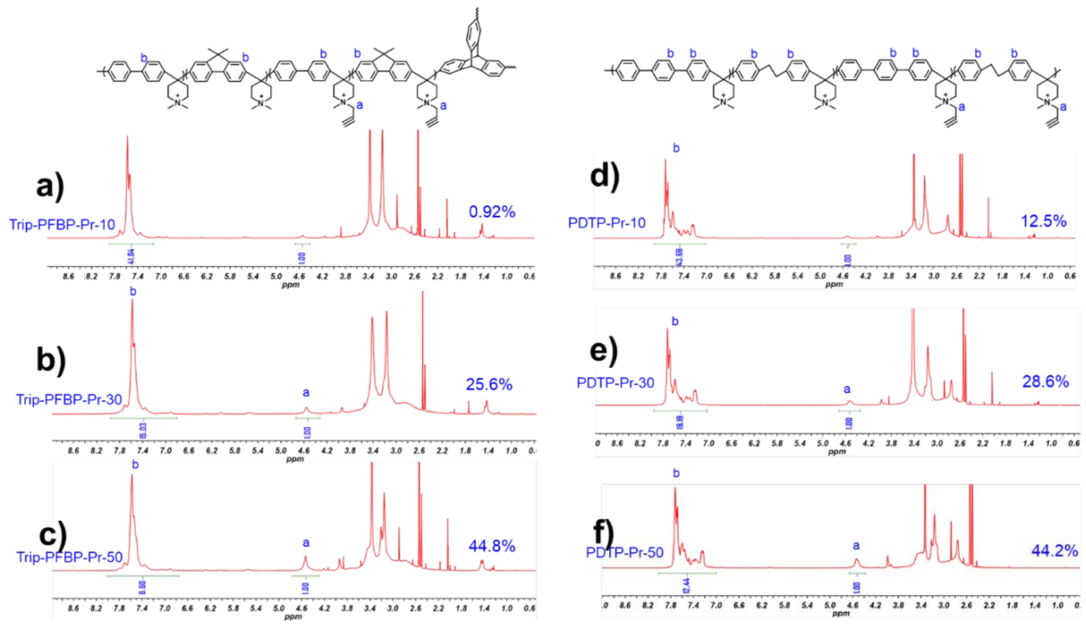

Figure S2. ^{1}H NMR spectra of Trip-PFBP-m and PDTP-Pr-m (m=10, 30, 50) polymers using DMSO-d6 as the solvent. a) Trip-PFBP-Pr-10, b) Trip-PFBP-Pr-30, c) Trip-PFBP-Pr-50, d) PDTP-Pr-10, e) PDTP-Pr-30, and f) PDTP-Pr-50.

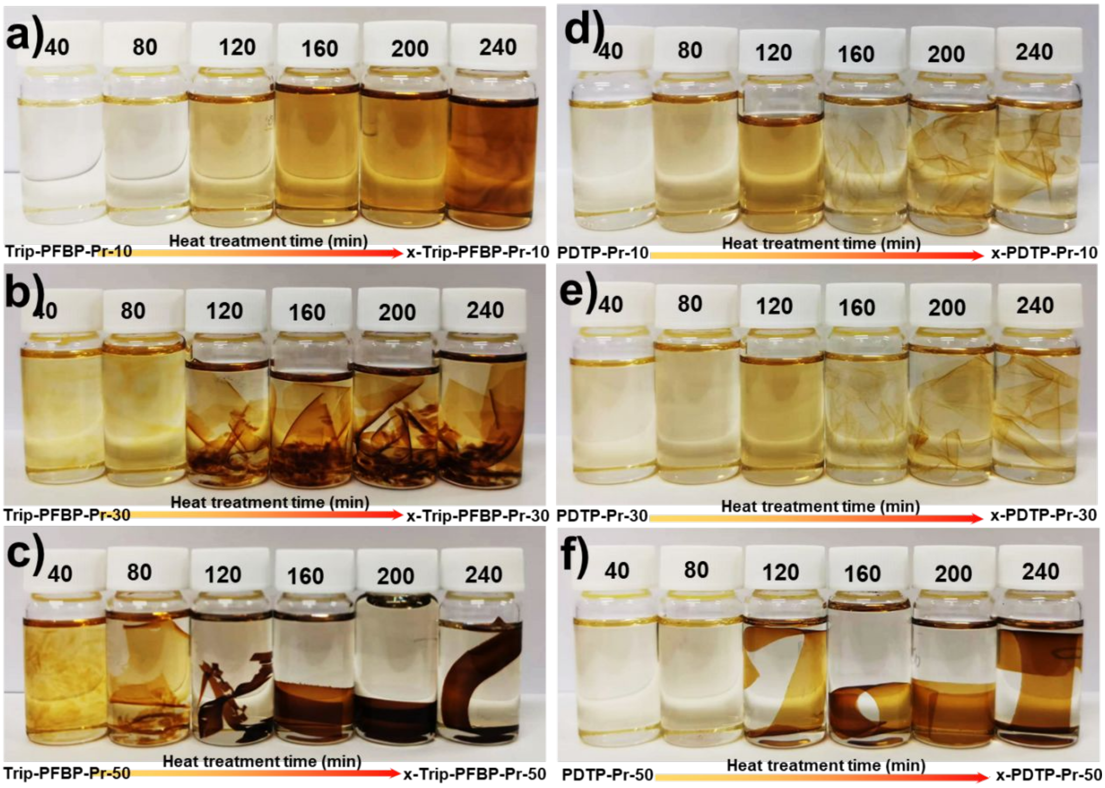

Figure S3. The solubility of Trip-PFBP-m and PDTP-Pr-m membranes in DMSO after thermal treatment for different times. a) Trip-PFBP-Pr-10, b) Trip-PFBP-Pr-30, c) Trip-PFBP-Pr-50, d) PDTP-Pr-10, e) PDTP-Pr-30, and f) PDTP-Pr-50.

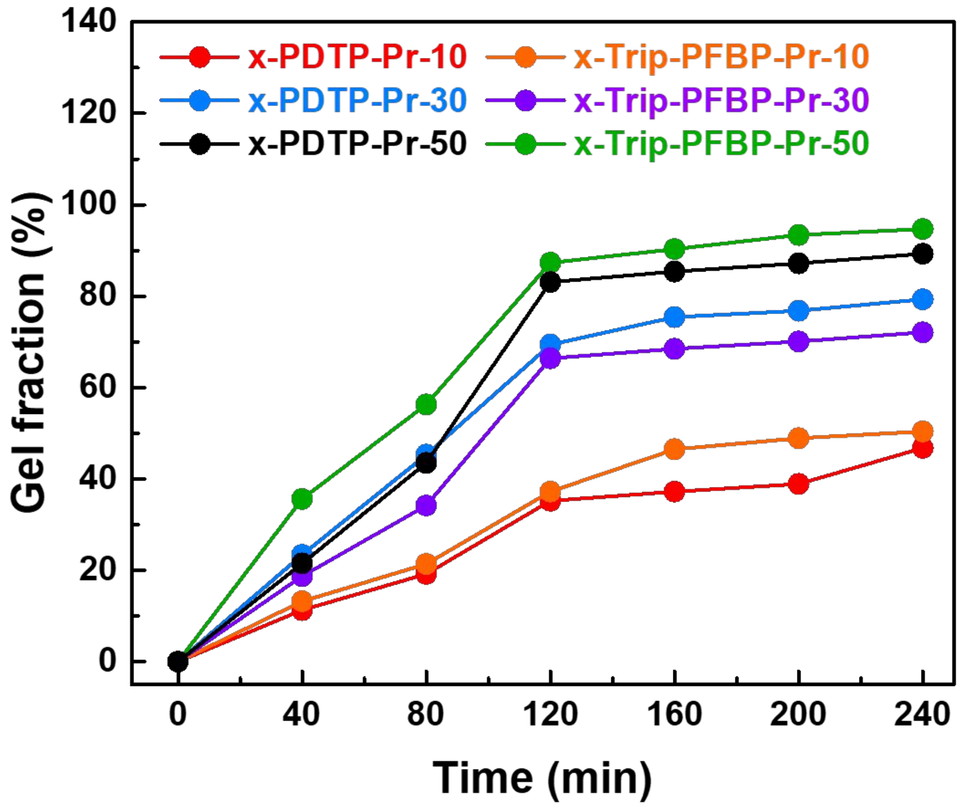

Figure S4. The gel fractions of Trip-PFBP-m and PDTP-Pr-m membranes with different thermal treatment times.

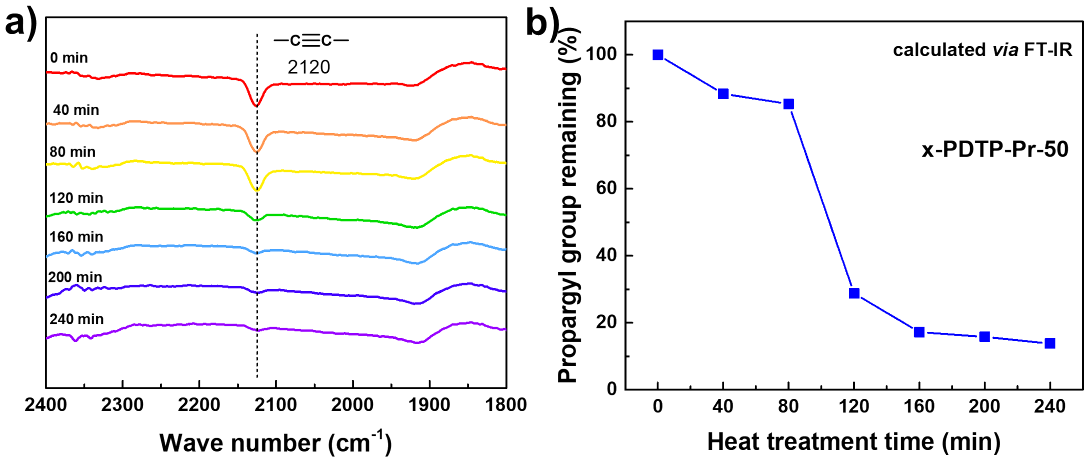

Figure S5. The crosslinking degree of x−PDTP-Pr-50 membranes with different thermal treatment times. a) FT-IR spectra of x-PDTP-Pr-50 with different thermal treatment times. b) The propargyl group remaining of the x-PDTP-Pr-50 membrane with different thermal treatment times calculated by integration of propargyl groups in the FT-IR spectra.

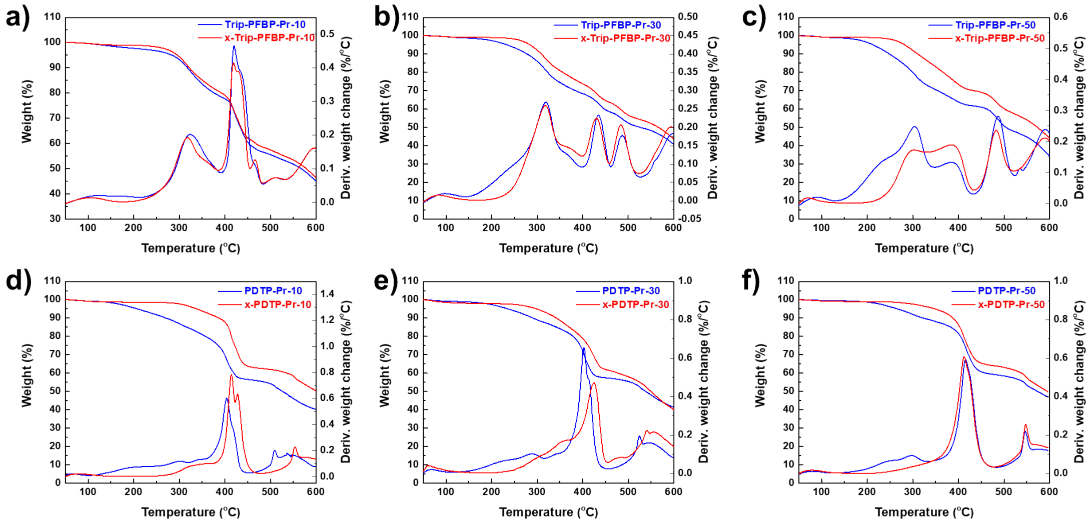

Figure S6. The thermal stability of membranes before and after thermal treatment. a) Trip-PFBP-Pr-10 and x-Trip-PFBP-Pr-10, b) Trip-PFBP-Pr-30 and x-Trip-PFBP-Pr-30, c) Trip-PFBP-Pr-50 and x-Trip-PFBP-Pr-50, d) PDTP-Pr-10 and x-PDTP-Pr-10, e) PDTP-Pr-30 and x-PDTP-Pr-30, and f) PDTP-Pr-50 and x-PDTP-Pr-50. Test conditions: under N_2 atmosphere from 50 to 600°C.

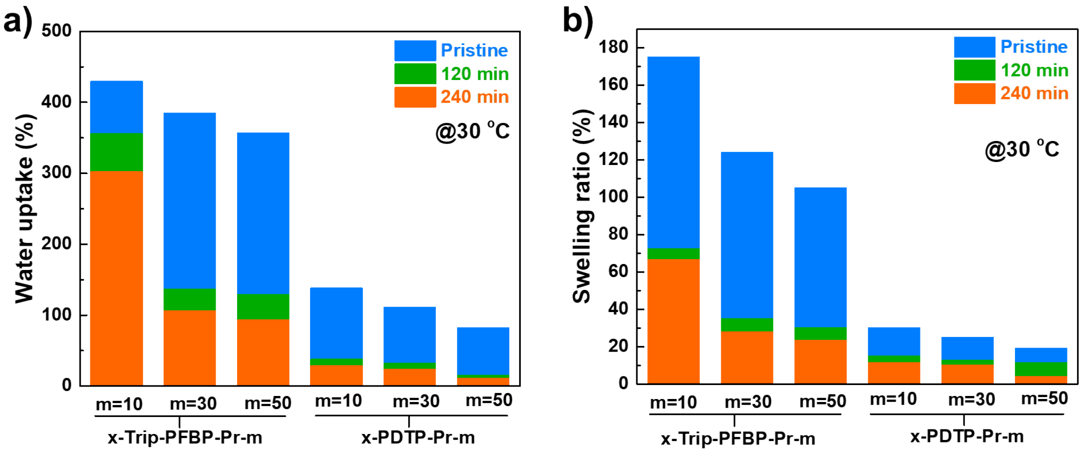

Figure S7. The water uptake and swelling ratio of crosslinked x-PDTP-Pr-x and x-Trip-PFBP-Pr-x membranes. a) The water uptake and b) swelling ratio of x-PDTP-Pr-m and x-Trip-PFBP-Pr-m membranes in OH^- form at 30 °C after thermal treatment of 0, 120, and 240 min.

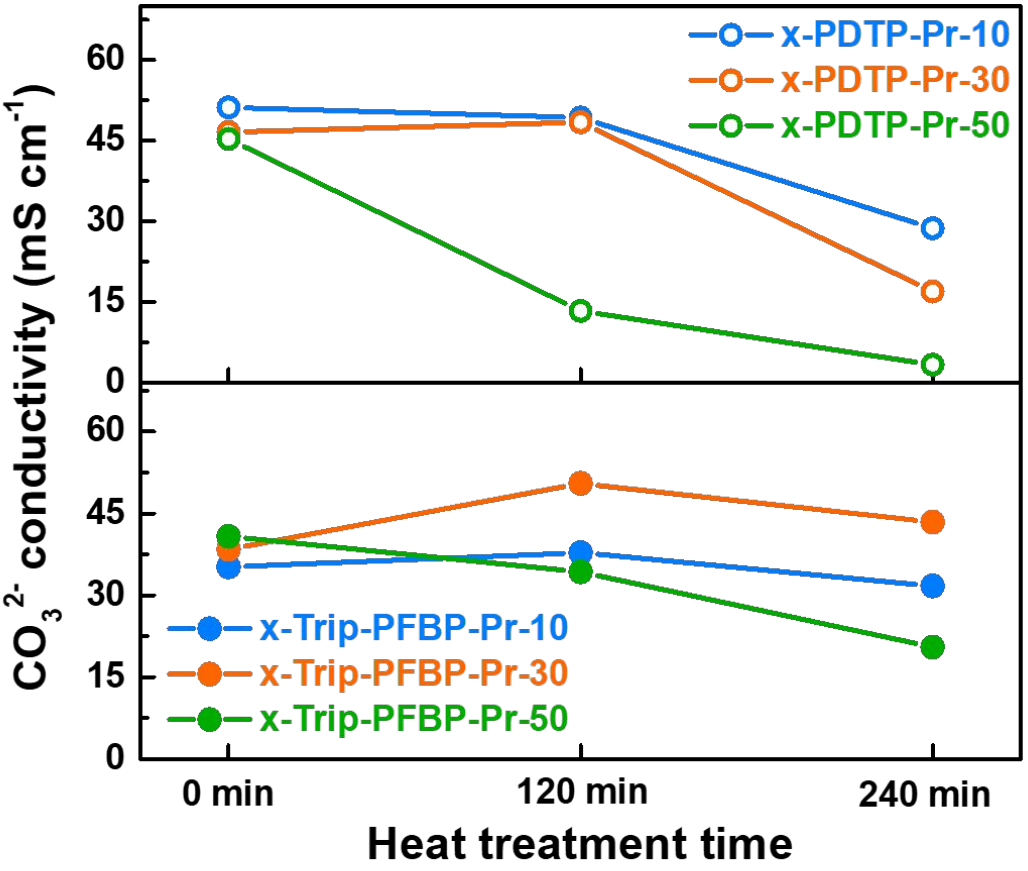

Figure S8. The carbonate conductivity of x-PDTP-Pr-m and x-Trip-PFBP-Pr-m membranes at 30°C after thermal treatment for 0, 120, and 240 min.

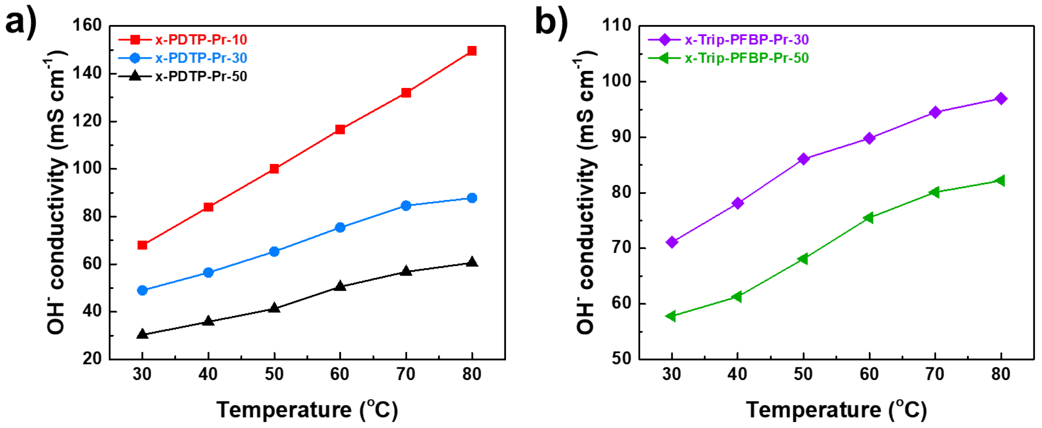

Figure S9. Hydroxide conductivity of a) x-PDTP-Pr-m and b) x-PFBP-Pr-m membranes after thermal treatment of 120 min.

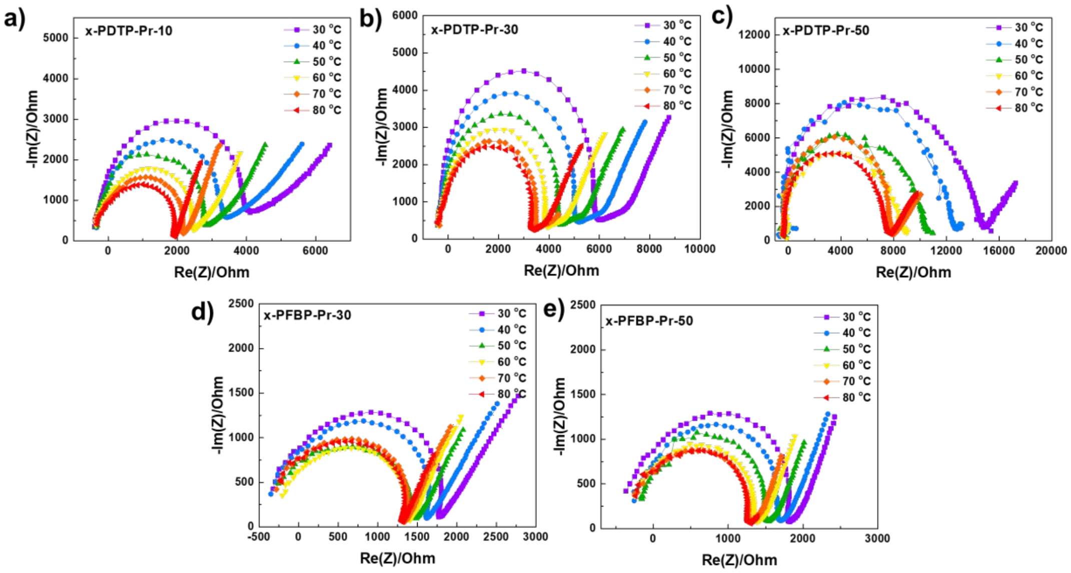

Figure S10. Ohmic resistance of a) x-PDTP-Pr-m and b) x-PFBP-Pr-m as a function of temperatures

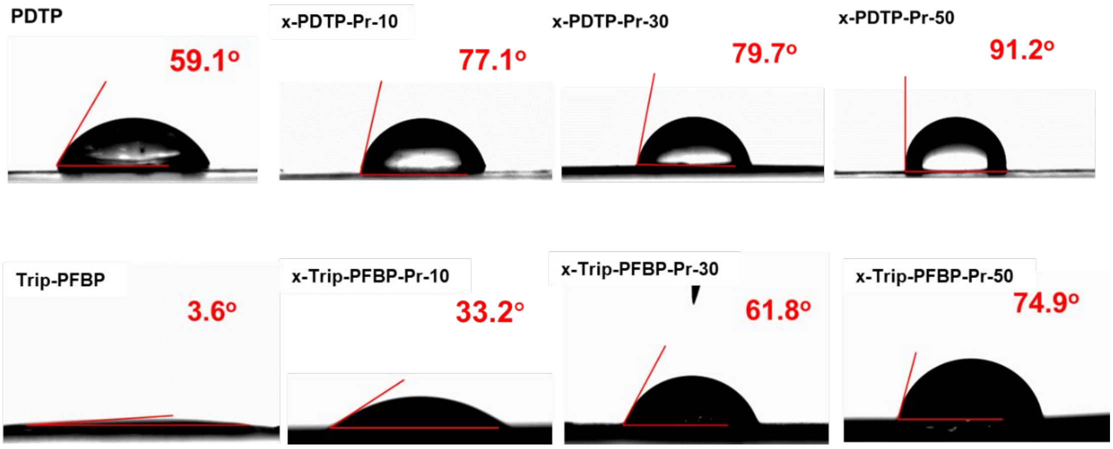

Figure S11. The static water contact angles of reference PDTP, Trip-PFBP and crosslinked x-PDTP-Pr-m, and x-Trip-PFBP-Pr-m membranes.

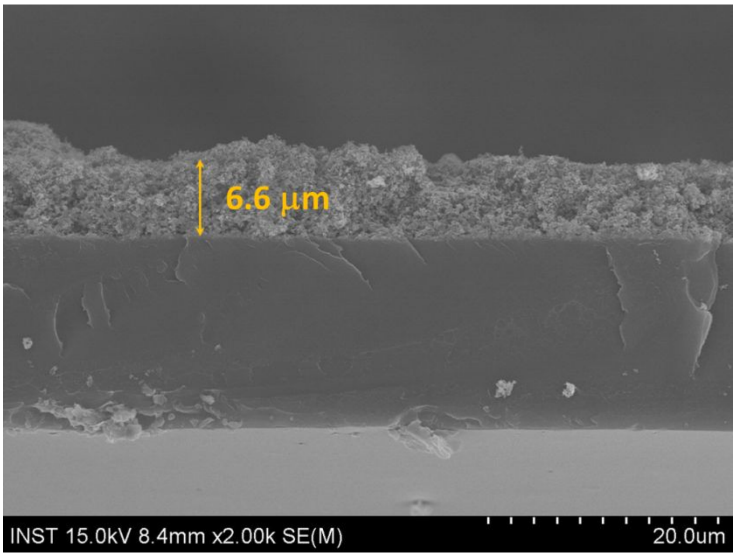

Figure S12. The SEM cross-sectional image of the MEA for the peeling-off measurement.

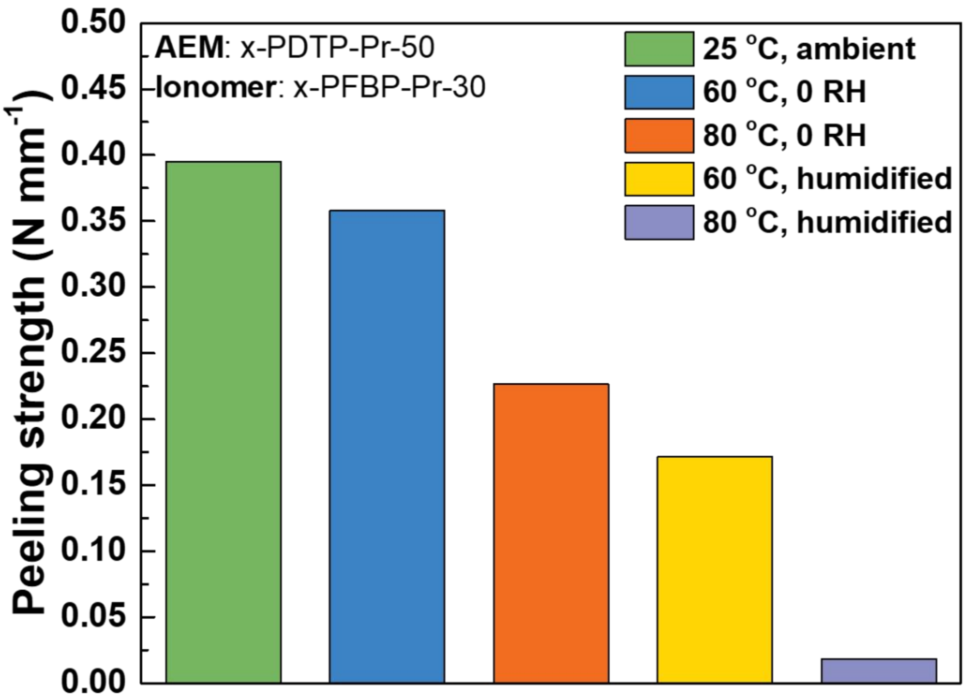

Figure S13. The peeling strength of x-PDTP-Pr-50&x-Trip-PFBP-Pr-30-based MEA at different temperatures and humidities.

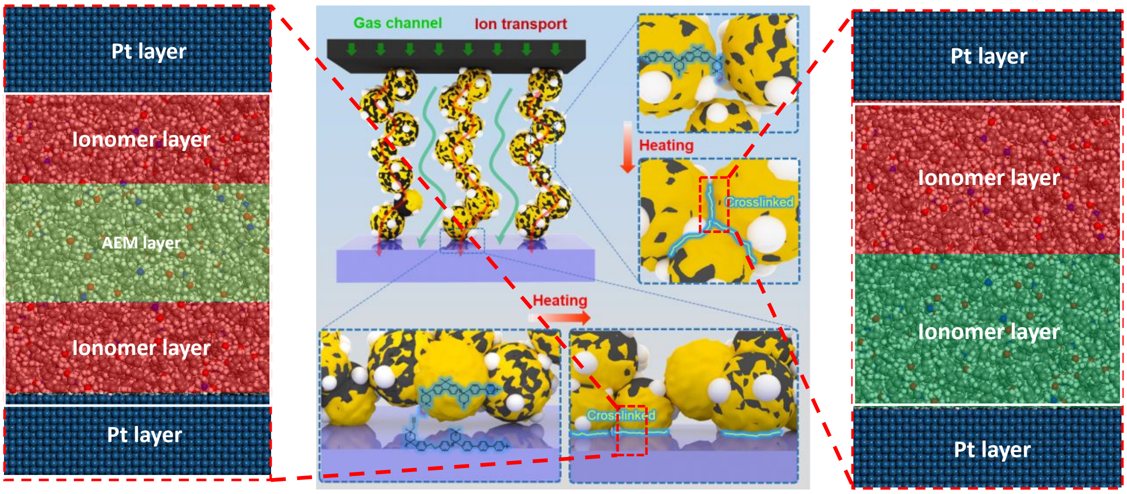

Figure S14. Atomistic models of the ionomer layer and AEM layer for MD simulations.

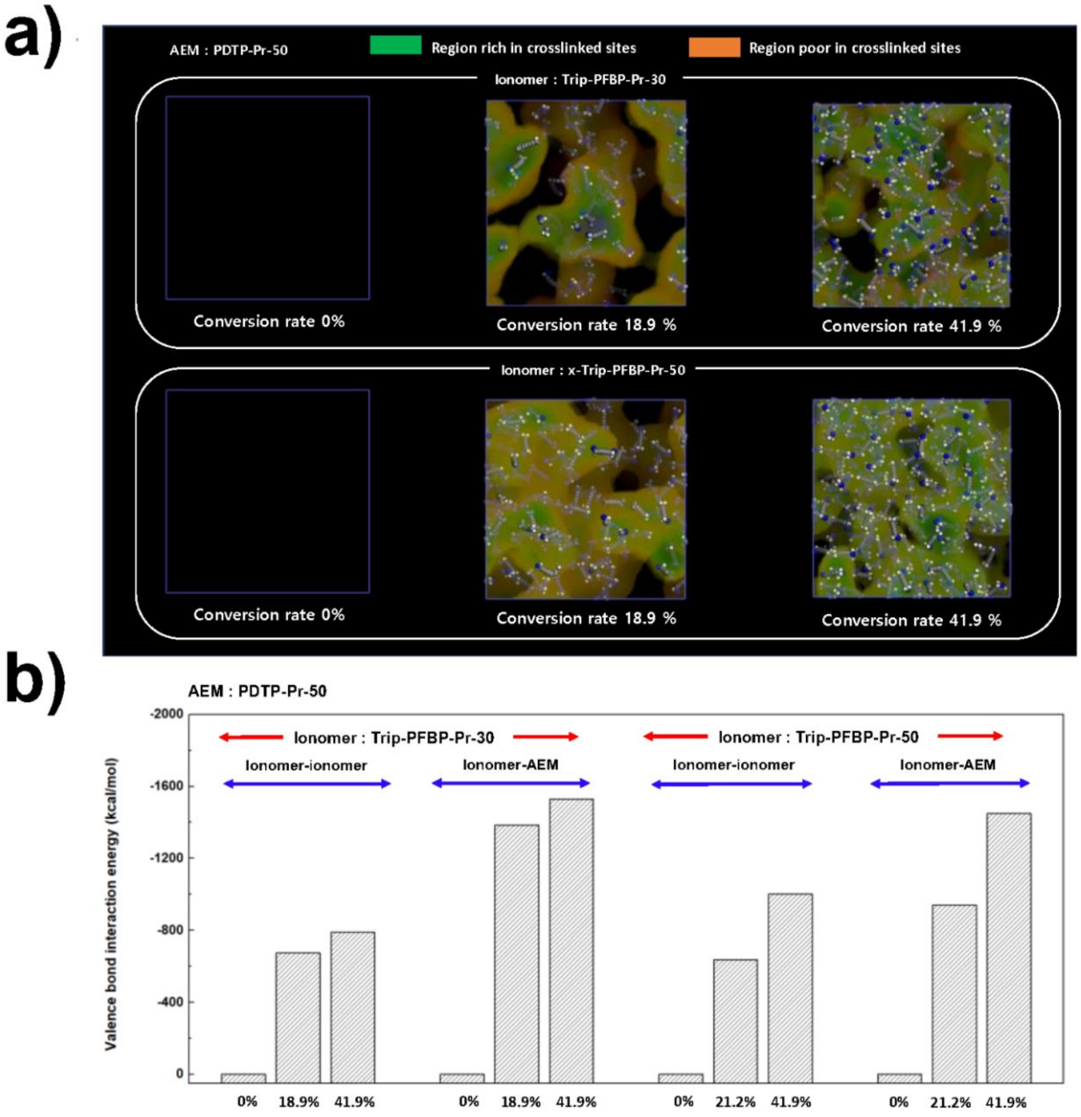

Figure S15. Crosslinked models *via* MD simulation and the interaction energies between the ionomer and AEM layers, as well as the ionomer layers themselves. a) In the crosslinked models, the density fields of the crosslinked sites are displayed in orange and green colors, which indicate the poorer and richer regions of the crosslinked sites, respectively. Also, atoms in the crosslinked site are displayed and the other atoms are hidden for clarity. As the models with a conversion rate of 0% indicate the non-crosslinked models, there is no density field inside the model, and empty boxes are displayed. b) The energies of valence bonding interactions were calculated using the difference of the valence bonding energy contribution in the potential energy of the fully combined layer model (ionomer layer + AEM layer) and the summation of the valence energy contribution of each layer.

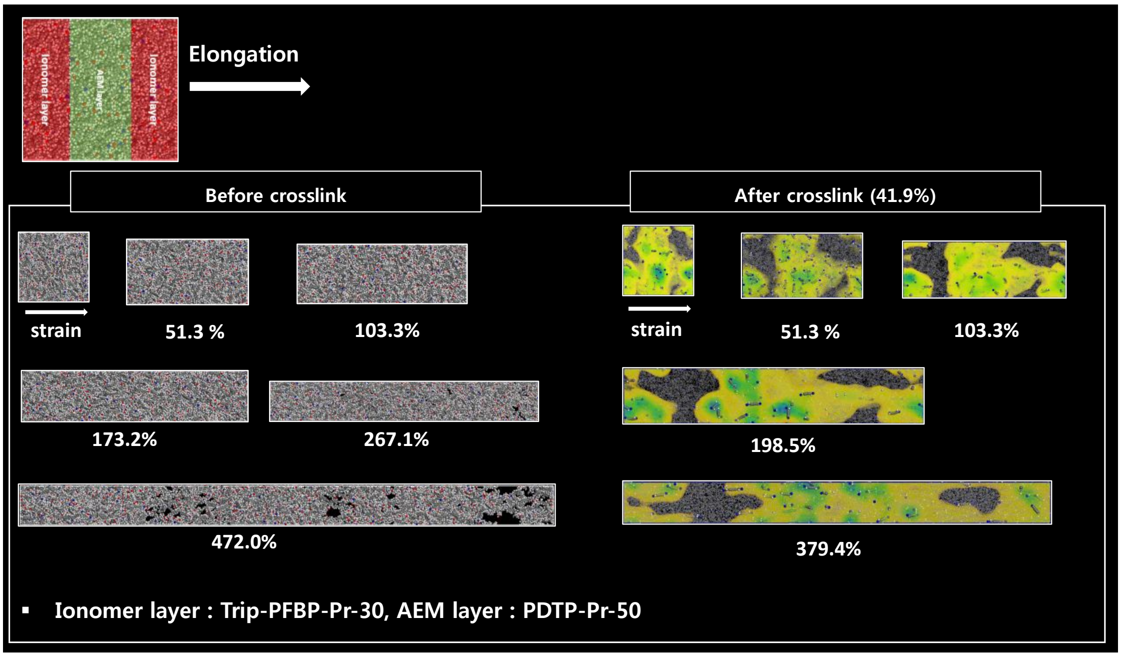

Figure S16. MD simulation of the elongation behaviors before and after crosslinking reaction. The non-crosslinked model shows cracks between the ionomer layer and the AEM layer and shows a much more flexible and stretched structure in the elongation simulation. On the other hand, the crosslinked model shows no crack between the ionomer layer and the AEM layer and shows a rigid structure in the elongation simulation. As shown in the density field of the crosslinked sites, the polymer chains and layers clump together due to the covalent bond after crosslinking, which can enhance their interactions and prevent detachment of the catalyst layer from the AEM.

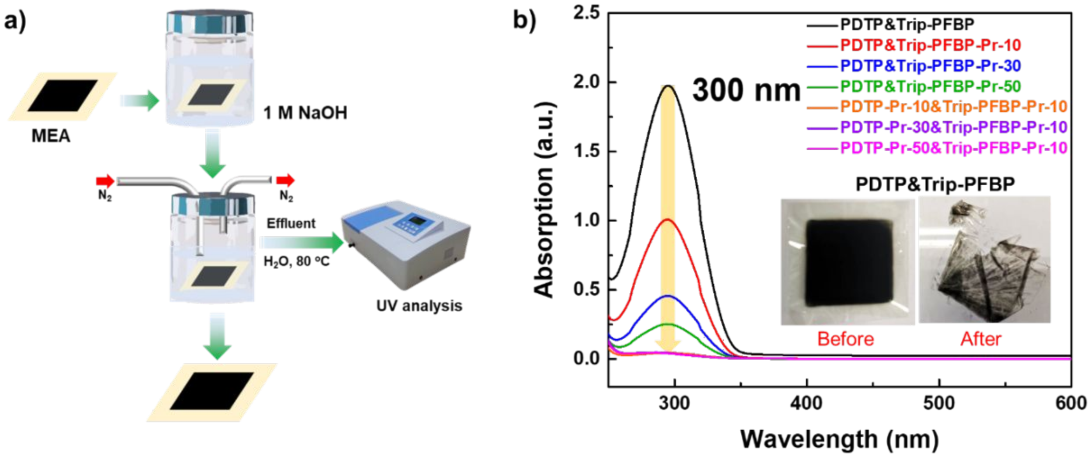

Figure S17. The catalyst layer stability measurement. a) The schematic diagram of ionomer effluent from MEA after treatment in 1 M NaOH solution at room temperature for 24 h and in deionized water at 80°C for 24 h. b) The UV absorption of the effluent from MEA. The adsorption in 300 nm is associated with aromatic units from ionomers.

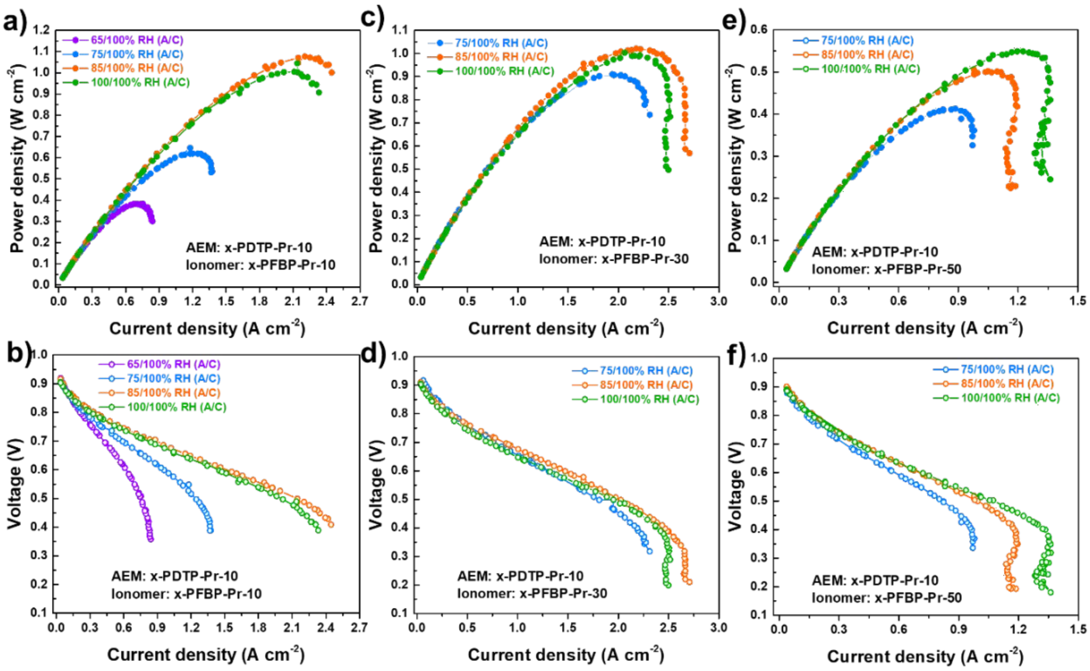

Figure S18. The fuel cell performance of x-PDTP-Pr-10 (25±2 μm) AEM-based AMEFC with different ionomers and different operation humidities. a) The polarization curves and b) the power density of x-Trip-PFBP-Pr-10 ionomer-based AEMFC. c) The polarization curves and d) power density of x-Trip-PFBP-Pr-30 ionomer-based AEMFC. e) The polarization curves and f) power density of x-Trip-PFBP-Pr-50 ionomer-based AEMFC.

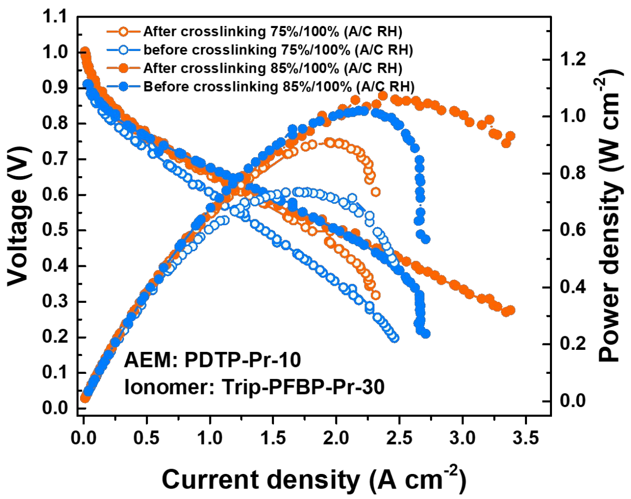

Figure S19. The polarization curves and power density of the MEA (AEM: PDTP-Pr-10; ionomer: Trip-PFBP-Pr-30) before and after crosslinking. Test conditions: cell temperature of 80°C, anode/cathode (A/C) relative humidity (RH) of 75%/100% or 85%/100%, A/C flowrate of H_2/O_2 1000/1000 mL min^{-1}, anode catalyst loading amount of 0.39 mgPtRu cm^{-2}, cathode catalyst loading amount of 0.26 mgPt cm^{-2}.

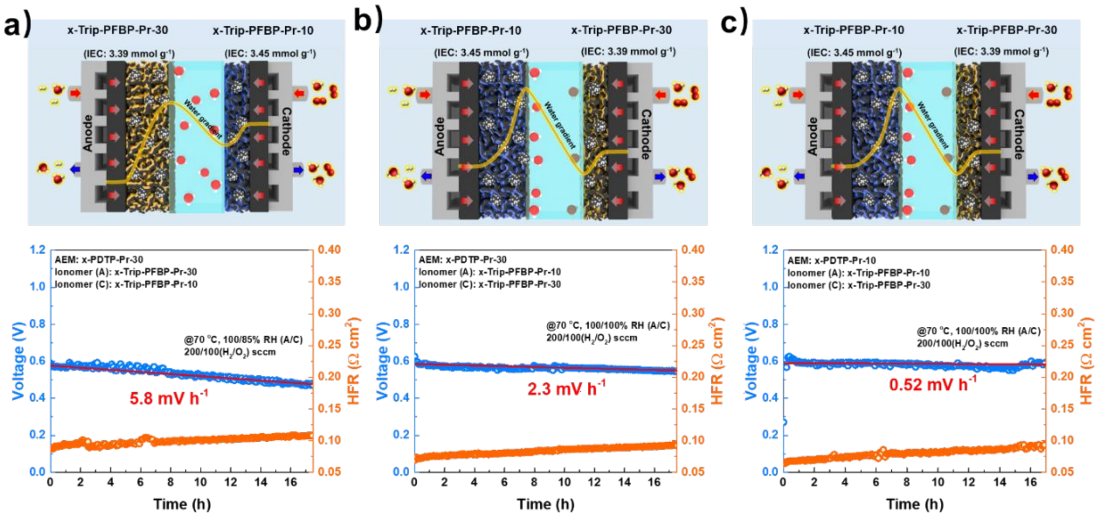

Figure S20. The in-situ durability of x-PDTP-Pr-30 (25±2 μm) AEM-based AMEFC with an asymmetric ionomer strategy. a) x-Trip-PFBP-Pr-30 as anode ionomer and x-Trip-PFBP-Pr-10 as cathode ionomer, b) x-Trip-PFBP-Pr-10 as anode ionomer and x-Trip-PFBP-Pr-30 as cathode ionomer, c) x-Trip-PFBP-Pr-10 as anode ionomer and x-Trip-PFBP-Pr-30 as cathode ionomer with replacement of the x-PDTP-Pr-30 AEM with x-PDTP-Pr-10 AEM. Test conditions: current density of 0.6 A cm^{-2}, cell temperature of 70°C, A/C flowrate of H_2/O_2 200/100 mL min^{-1}, anode catalyst loading amount of 0.4 mg_{Pt} cm^{-2}, cathode catalyst loading amount of 0.4 mg_{Pt} cm^{-2}.

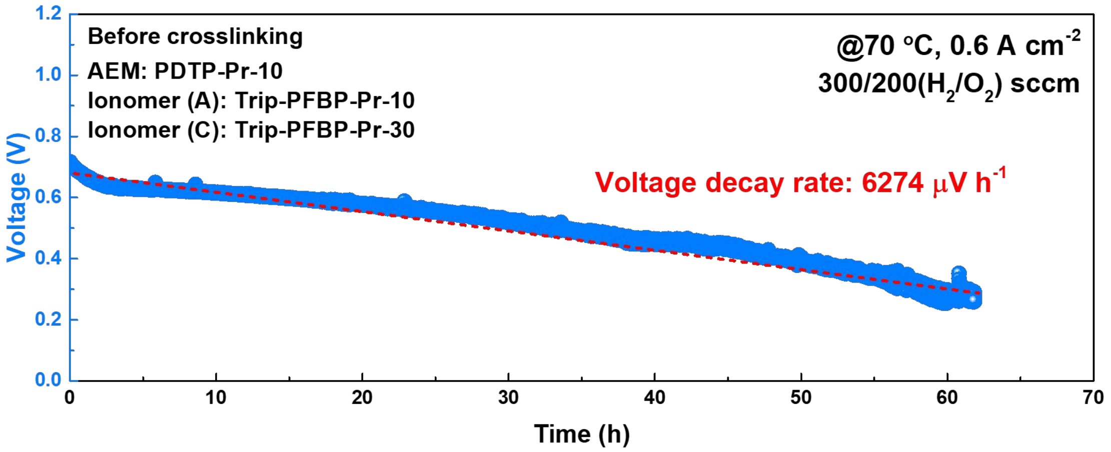

Figure S21. The in-situ durability test of PDTP-Pr-10 AEM (before crosslinking)-based AEMFC at 0.6 A cm^{-2} with Trip-PFBP-Pr-10 (before crosslinking) as anode ionomer and Trip-PFBP-Pr-30 (before crosslinking) as cathode ionomer. Test conditions: cell temperature of 70°C, A/C RH of 94/100%, A/C flowrate of H_2/O_2 300/200 mL min^{-1}, anode catalyst loading of 0.4 mg_{Pt} cm^{-2}, cathode catalyst loading of 0.4 mg_{Pt} cm^{-2}.

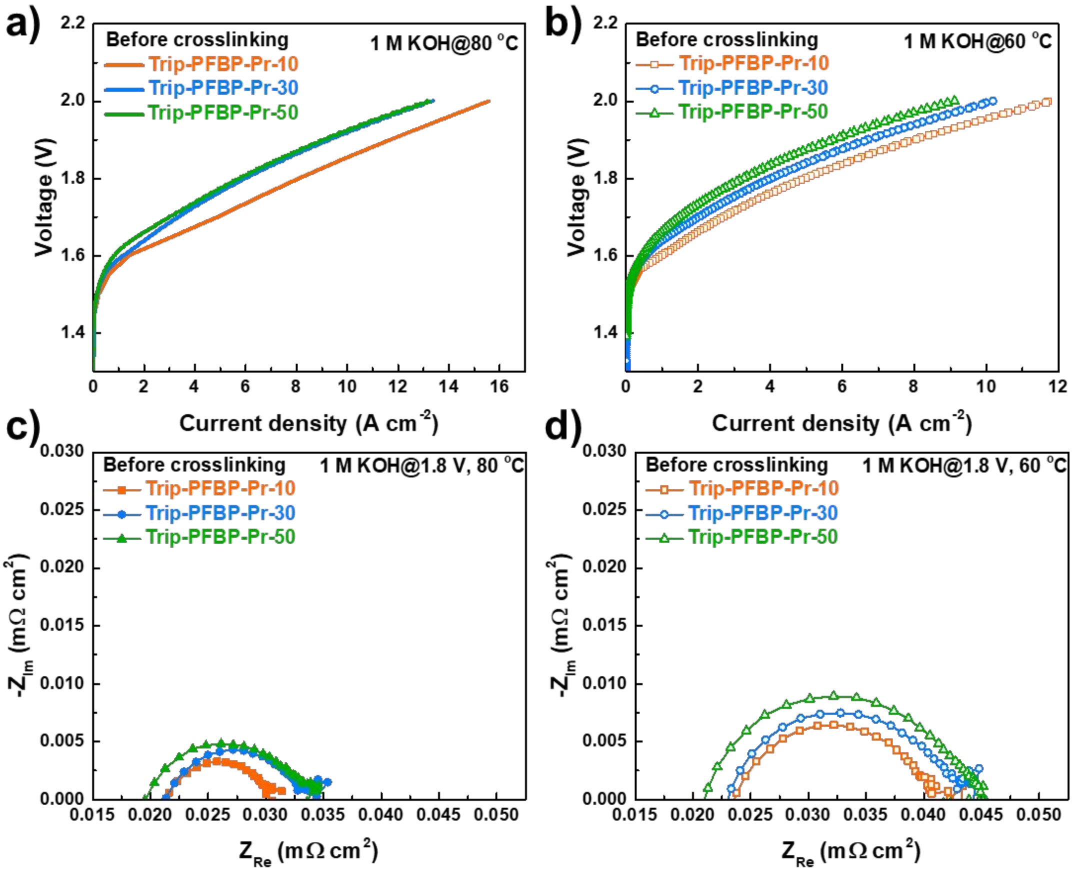

Figure S22. Linear scan voltammograms (LSV) of PDTP-Pr-10 (before crosslinking) AEM (20 μm)-based AEMWE with different ionomers at a) 80°C and b) 60℃ in 1 M KOH solution. Potentiostatic electrochemical impedance spectroscopy (PEIS) of PDTP-Pr-10 (before crosslinking) AEM (20 μm)-based AEMWE with different ionomers at a) 1.8 V, 80°C and b) 1.8 V, 60℃ in 1 M KOH solution.

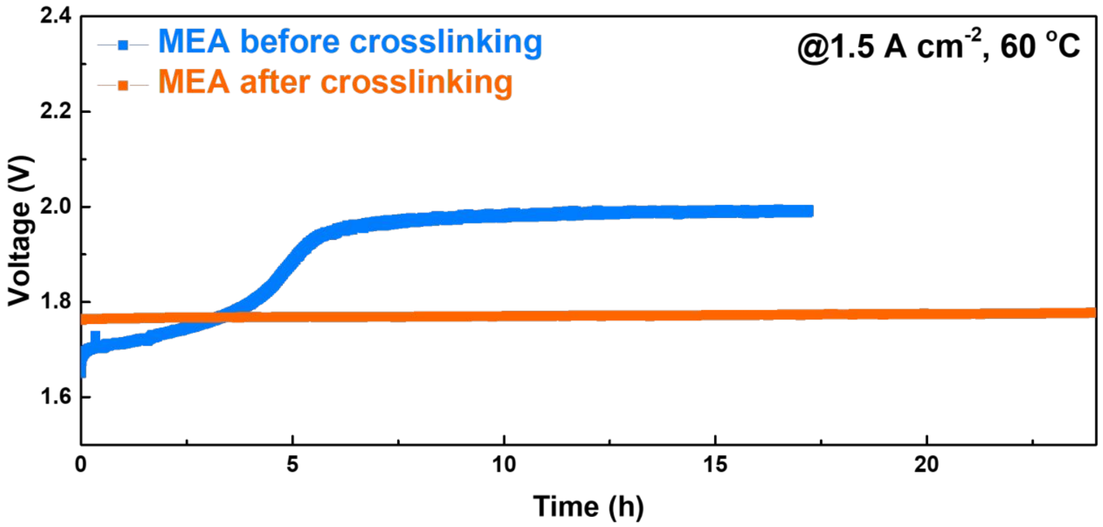

Figure S23. The in-situ durability of x-PDTP-Pr-10 AEM (after crosslinking, 25 μm) and PDTP-Pr-10 AEM (before crosslinking, 25 μm)-based AEMWEs under 60°C at 1 M KOH solution and a current density of 1.5 A cm^{-2}. Test conditions: alkali flow rate of 36 mL min^{-1}, anode catalyst of IrO_2 (2.0 mg cm^{-2}), cathode catalyst of PtRu/C (0.7 mg cm^{-2}).

Table S1. Optical images of the catalyst-coated membranes with different compositions before and after ionomer effluent measurements

AEMs / AEIs	x-Trip-PFBP-Pr-10		x-Trip-PFBP-Pr-30		x-Trip-PFBP-Pr-50	
	Before test	After test	Before test	After test	Before test	After test
PDTP	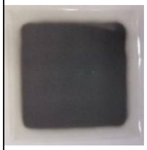	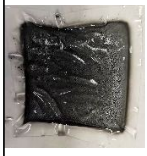	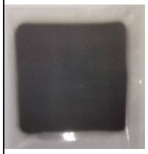	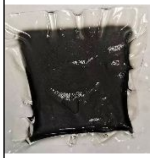	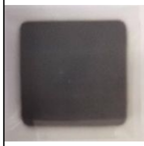	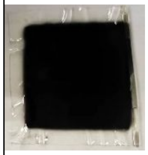
x-PDTP-Pr-10	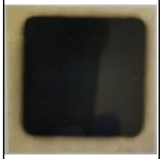	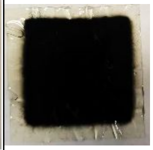	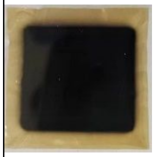	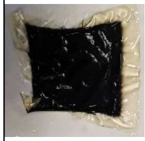	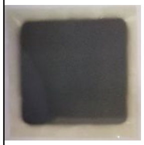	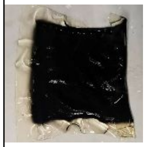
x-PDTP-Pr-30	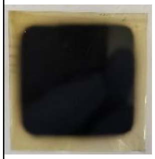	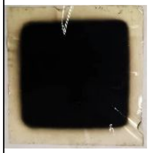	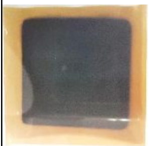	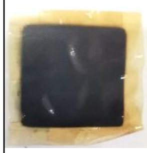	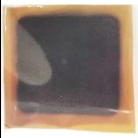	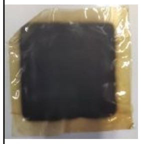
x-PDTP-Pr-50	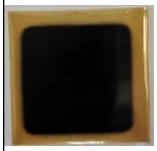	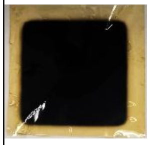	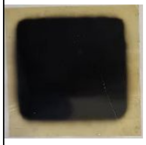	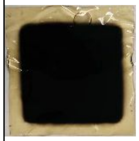	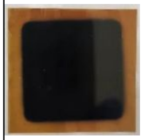	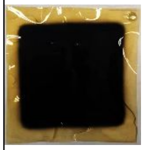
