# Supplementary material for: Stabilizing the Catalyst Layer for Durable and High Performance Alkaline Membrane Fuel Cells and Water Electrolyzers
Source: ACS Cent Sci. 2024 Feb 15;10(3):603–14. doi: 10.1021/acscentsci.3c01490 (PMC10979504; doi:10.1021/acscentsci.3c01490)
Supplement: Supplementary file 2 — oc3c01490_si_002.pdf [file oc3c01490_si_002.pdf]

oc-2023-014909.R1

Name: Peer Review Information for "Stabilizing the catalyst layer for durable and high performance alkaline membrane fuel cells and water electrolyzers"

First of Reviewer Comments

Reviewer: 1

Comments to the Author

The authors present an approach to enhance the stability and performance of catalyst layers in alkaline membrane fuel cells and water electrolyzers. This is achieved through an in-situ cross-linking strategy that improves interactions within the catalyst layer and between the catalyst layer and anion exchange membranes. The paper provides detailed insights into the synthesis of cross-linked polymers, their physical and electrochemical characterization, and application effectiveness. Emphasis is placed on the significant improvement in adhesion strength and operational stability, marking a notable advancement in the field. However, there are still some comments to be addressed.

1. Since different ionomers and membranes were selected for the test conditions, it is necessary to do EIS analyses under the test conditions. This would provide valuable insights into the electrochemical properties of the catalyst layer, especially in understanding the impedance changes due to cross-linking. It would be beneficial for the authors to include EIS analysis at different frequencies to comprehensively evaluate the electrochemical behavior of the catalyst layer in both cross-linked and non-crosslinked states.

2. The article includes a peel-off test to evaluate the adhesion strength of the catalyst layer. A suggestion to improve this analysis is to perform the peel-off test under varying environmental conditions, such as different temperatures or humidities. This would provide insights into the robustness of the adhesion under conditions that more closely mimic practical operation conditions.

3. I recommend the authors test hydroxide ion (OH^-) conductivity, which is crucial in fuel cells and water electrolyzers due to its role as the primary charge carrier in alkaline environments. The authors measured carbonate ion (CO_3^{2-}) conductivity, which does not seem directly relevant to the alkaline fuel cell and water electrolyzer applications.

4. In the last paragraph on page 11, the author made a labeling error; "Figure S10b" should be corrected to "Figure S13b".

5. The article doesn't specify the exact electrolyte used in the stability tests. Also, the authors did not provide a detailed explanation for how the refreshment of the MEA contributes to prolonged

stability. Given this, I suggest the authors clarify the type of electrolyte used in their stability tests and provide a more detailed explanation or hypothesis about the mechanism by which MEA refreshment extends the operational stability of the system.

6. I suggest that the authors compare the performance and durability of thermally treated and non-treated systems in fuel cells and water electrolysis tests.

Reviewer: 2

Comments to the Author

In this paper, a facile approach to enhance the stability of the catalyst layer by increasing the interactions between the catalyst layer and AEM is proposed. Alkyne groups are introduced in AEM and ionomer, which could form cross-linking by heating. Proper cross-linking would improve the dimensional stability of AEM and the connection between AEM and catalyst layer leading to excellent operational stability in AEMFC and AEMWE. However, there are some questions that should be solved before published.

1. The text writes that "As heat treatment time increased, the strength of the alkyne absorption peak decreased, suggesting consumption of the propargyl group during the heating process." But triple-bonds are remained in mechanism diagram along with the consumption of alkyne. (Fig.1, Fig.S1) In other words, the crosslinking was done in the wrong way.

2. What's the difference between changing the acetylene grafting ratio and crosslinking time in MEA preparation? Which is better?

3. Whether cross-linking between ionomers would increase the mass transfer resistance? And what's the evidence?

4. "Meanwhile, the weak connection between the catalyst layer and AEM results in detachment of the catalyst layer, as displayed in the inset pictures in Figure 1a.To evaluate the interactions with the catalyst layer, the peeling strength of the catalyst layer was measured using a peel-off test." "The higher peeling strength of MEA suggests stronger interactions between catalyst layers". What is the thickness of the catalyst layer tested? Whether the test is between catalyst layers or between catalyst layers and AEM? If between catalyst layers, why do the interactions between the same ionomers change as the substrate AEM changes in Fig. 2c?

5. "The oxygen bubbles generated in the anode will crash the catalyst causing the dispersion or detachment of the catalyst due to poor connection of catalyst layers, especially at a high current density (see Figure 5a). Therefore, stabilizing the catalyst layer is crucial for the long-term operation of AEMWEs." There is still poor connection in anode used in AEMWE.

6. There are still syntax errors that need to be corrected.

Author's Response to Peer Review Comments:

January 13, 2023

To Professor Editor

Senior Editor

ACS Central Science

Subject: Submission of the revised manuscript, Manuscript ID: oc-2023-014909 to ACS Central Science

Dear Professor Editor

Enclosed please find our revised manuscript entitled, “Stabilizing the catalyst layer for durable and high-performance alkaline membrane fuel cells and water electrolyzers”, for consideration as a research article in ACS Central Science. This work includes the authors of Chuan Hu, Hyun Woo Kang, Seung Won Jung, Xiaohua Zhang, Young Jun Lee, Na Yoon Kang, Chi Hoon Park, and the corresponding author of Young Moo Lee

We appreciate you and the reviewers for the time and efforts to evaluate our work. We tried our best to answer all the questions and comments and made point-by-point responses to the comments. All the changes are highlighted in yellow in the revised manuscript. The followings are the improvements that we made in the main text.

- The hydroxide conductivity of the membranes has been tested.
- The peeling strength at different temperatures and humidity conditions has been tested.
- The performance and durability of thermally treated and non-treated systems in fuel cells and water electrolysis have been tested.
- The syntax errors have been corrected.

We submit this manuscript to ACS Central Science because we believe it is of broad interest and will impact the development of anion exchange membrane fuel cells and water electrolyzers.

This manuscript has been approved by all authors for publication. The manuscript has not been submitted or considered for publication elsewhere.

If you have any questions or require any more information, please feel free to contact us by email.

Thank you.

Sincerely,

Young Moo Lee, PhD

Distinguished Professor

Response to reviewers

Manuscript title: Stabilizing the catalyst layer for durable and high performance alkaline membrane fuel cells and water electrolyzers

Manuscript ID: oc-2023-014909

Authors: Chuan Hu, Hyun Woo Kang, Seung Won Jung, Xiaohua Zhang, Young Jun Lee, Na Yoon Kang, Chi Hoon Park, Young Moo Lee*

We appreciate the Editor and Reviewers for their time and efforts to evaluate our work. We tried our best to answer all the questions and comments and made point-by-point responses. All the changes are highlighted in yellow in the revised version.

Response to Editor

SYNOPSIS MISSING: The synopsis should be no more than 200 characters (including spaces) and should reasonably correlate with the TOC graphic. The synopsis is intended to explain the importance of the article to a broader readership across the sciences. Please place your synopsis in the manuscript file after the TOC graphic, and label it as "Synopsis."

Response: Thank you. We have added and highlighted the synopsis in the revised manuscript.

Prior to submitting your revision, please also be sure to address the formatting issues listed above the reviewer comments. Further information regarding press, hero images, etc. is included in the attached 'Author Checklist' document. Please note that certain non-scientific needs are required prior to acceptance.

Response: Thank you. All the comments have been answered.

Funding Sources: Authors are required to report ALL funding sources and grant/award numbers relevant to this manuscript. Enter all sources of funding for ALL authors relevant to this manuscript in BOTH the Open Funder Registry tool in ACS Paragon Plus and in the manuscript to meet this requirement. See http://pubs.acs.org/page/4authors/funder_options.html for complete instructions.

Response: Thank you. We have checked the funding sources.

Reviewer #1

The authors present an approach to enhance the stability and performance of catalyst layers in alkaline membrane fuel cells and water electrolyzers. This is achieved through an in-situ cross-linking strategy that improves interactions within the catalyst layer and between the catalyst layer and anion exchange membranes. The paper provides detailed insights into the synthesis of cross-linked polymers, their physical and electrochemical characterization, and application effectiveness. Emphasis is placed on the significant improvement in adhesion strength and operational stability, marking a notable advancement in the field. However, there are still some comments to be addressed.

Response: Thank you for your positive comments. We made a point-to-point response to your

following comments.

1. Since different ionomers and membranes were selected for the test conditions, it is necessary to do EIS analyses under the test conditions. This would provide valuable insights into the electrochemical properties of the catalyst layer, especially in understanding the impedance changes due to cross-linking. It would be beneficial for the authors to include EIS analysis at different frequencies to comprehensively evaluate the electrochemical behavior of the catalyst layer in both cross-linked and non-crosslinked states.

Response: Thank you for your suggestion. The EIS curves of the AEMWEs using Trip-PFBP-Pr-m ionomers with non-crosslinked states have been tested from 100 Hz to 200 kHz and added in **Figure R1** (Figure S21c and S21d in the revised Supporting Information). The ionomers under non-crosslinked states show lower Ohmic resistance (0.0196-0.0215 Ω cm^2 in 1 M KOH, 1.8 V) compared with that of the ionomers with crosslinked structure (0.0227-0.028 Ω cm^2). The increased Ohmic resistance of the catalyst layers is associated with the hydrophobic crosslinking structures. Additionally, the charge transfer resistance of the catalyst layers after crosslinking increased from 0.0089-0.0149 Ω cm^2 to 0.0122-0.0223 Ω cm^2, suggesting that the crosslinked structure limits the charge transport of the catalyst layer. If the ionomers have a high crosslinking degree, the negative effect on resistance is more severe. Therefore, the crosslinking degree of the ionomers should be controlled at an appropriate level. In that case, we used x-PDTP-Pr-10 as AEM, x-PFBP-Pr-10, and x-PFBP-Pr-30 as ionomers. This part has been reorganized in the revised manuscript.

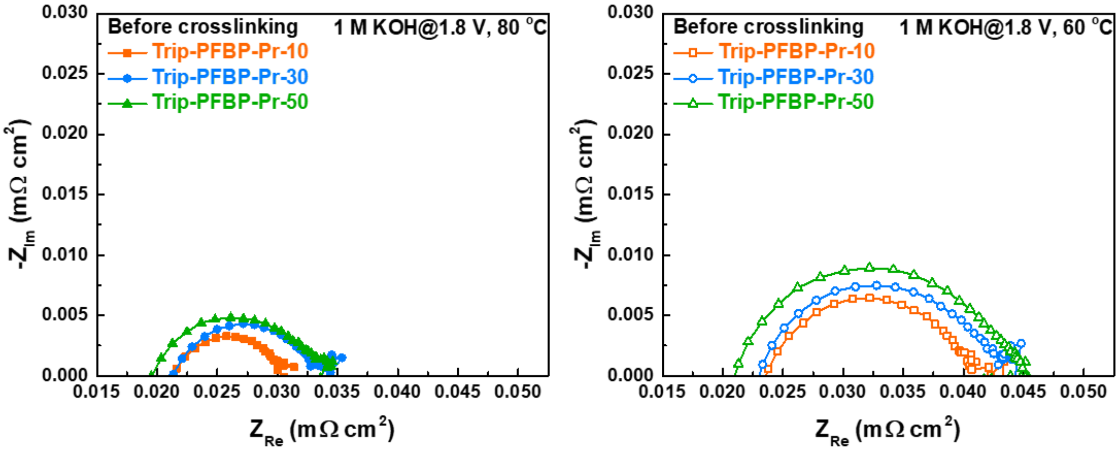

Figure R1. The potentiostatic electrochemical impedance spectroscopy (PEIS) of PDTP-Pr-10 (before crosslinking) AEM (20 μm)-based AEMWE with different ionomers at c) 1.8 V, 80°C and d) 1.8 V, 60°C in 1 M KOH solution.

2. The article includes a peel-off test to evaluate the adhesion strength of the catalyst layer. A suggestion to improve this analysis is to perform the peel-off test under varying environmental conditions, such as different temperatures or humidities. This would provide insights into the robustness of the adhesion under conditions that more closely mimic practical operation conditions.

Response: Thank you for your constructive suggestions. It is worthwhile to analyze the effect of temperature and humidity on the adhesion. We tried to measure the peeling strength of the MEA under 60°C, 80°C, 0 relative humidity (RH), and humidified conditions using x-PDTP-Pr-50 as

AEM, and x-Trip-PFBP-Pr-30 as ionomers. As shown in **Figure R2** (Figure S13 in the revised Supporting Information), the peeling strength of the MEA decreases with the increase in temperature. Additionally, the humidified environment conditions further impair the interaction of the catalyst layer, resulting in a lower peeling strength. This part has been reorganized in the revised manuscript.

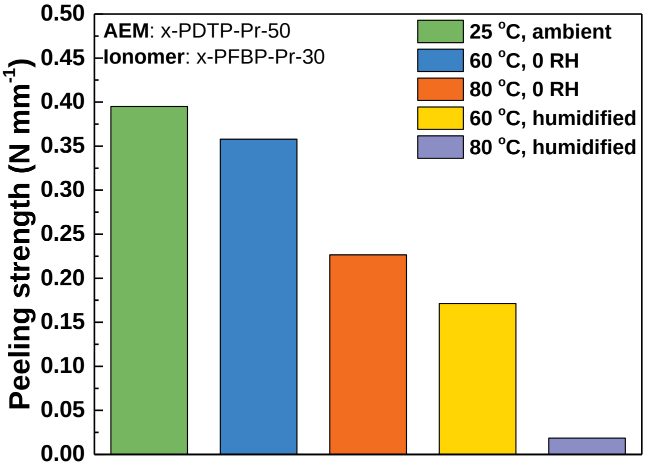

Figure R2. The peeling strength of x-PDTP-Pr-50&x-Trip-PFBP-Pr-30-based MEA at different temperatures and humidities.

Additionally, we added the following statement in the main text in page 11, lines 185-190.
"The effect of operation temperature and humidity on the peeling-strength was further investigated as shown in Figure S13. As the temperature increased to 80°C, the peeling strength of x-PDTP-Pr-50&x-Trip-PFBP-Pr-30-based MEA decreased from 0.395 to 0.226 N mm^{-1}. At humidified conditions, the peeling strength was further decreased to 0.018 N mm^{-1}, suggesting that the interaction between the catalyst layer and membrane is vulnerable at high temperature and wet conditions."

3. I recommend the authors test hydroxide ion (OH^-) conductivity, which is crucial in fuel cells and water electrolyzers due to its role as the primary charge carrier in alkaline environments. The authors measured carbonate ion (CO_3^{2-}) conductivity, which does not seem directly relevant to the alkaline fuel cell and water electrolyzer applications.

Response: Thank you for your suggestion. The OH^- conductivity and the EIS curves have been measured as shown in **Figures R3** and **R4** (Figures S9 and S10 in revised supporting information). x-Trip-PFBP-Pr-10 ionomer in OH^- form has a high-water uptake and swelling ratio which is not suitable for the conductivity measurement. The related discussion also has been added and highlighted in the revised manuscript in page 9 lines 153-159.

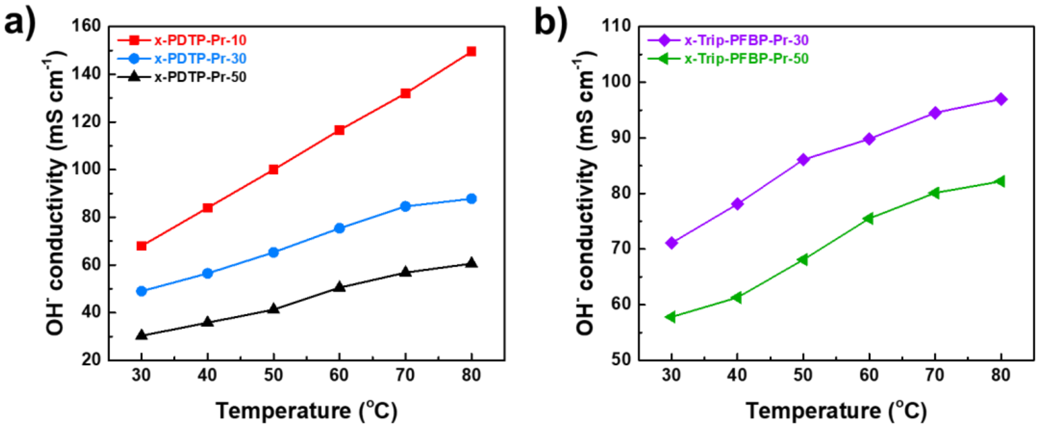

Figure R3. Hydroxide conductivity of a) x-PDTP-Pr-m and b) x-PFBP-Pr-m as a function of temperatures

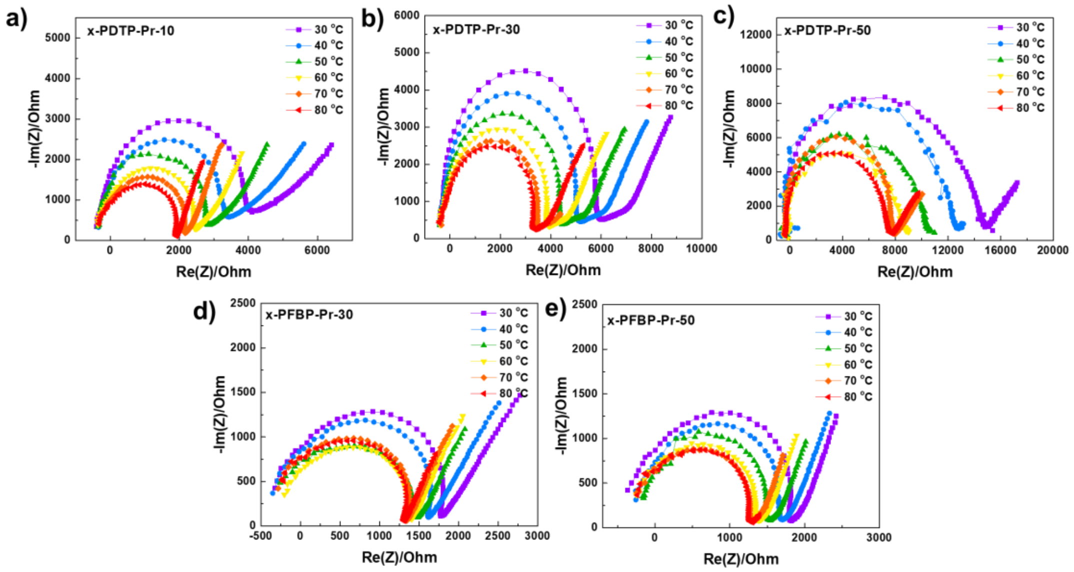

Figure R4. Ohmic resistance of a) x-PDTP-Pr-m and b) x-PFBP-Pr-m as a function of temperatures

4. In the last paragraph on page 11, the author made a labeling error; "Figure S10b" should be corrected to "Figure S13b".

Response: Thank you for pointing the out our typo. We have corrected it.

5. The article doesn't specify the exact electrolyte used in the stability tests. Also, the authors did not provide a detailed explanation for how the refreshment of the MEA contributes to prolonged stability. Given this, I suggest the authors clarify the type of electrolyte used in their stability tests and provide a more detailed explanation or hypothesis about the mechanism by which MEA refreshment extends the operational stability of the system.

Response: Thank you for your suggestion. During the AEMWE durability test, 1M KOH solution was used as an electrolyte at the anode side. On the cathode side, we applied a dried electrode strategy. In other words, we did not feed in liquid electrolytes at the cathode side. We have

reorganized this part and specified this information in the revised manuscript.

Page 15, lines 278-280

"During the test, the fuel cell was refreshed by immersing the MEA in an alkaline solution overnight and then washed with deionized water to remove residual alkali. Finally, the refreshed MEA was reassembled in the fuel cell station."

Page 15, lines 282-286

"After the replenishment process, the decreased voltage was recovered along with a decreased HFR, which suggests that the decreased voltage during the steady-state operation is probably caused by other reasons (e.g., carbonation or uneven water distribution) and not the chemical degradation of the membrane and ionomer."

6. I suggest that the authors compare the performance and durability of thermally treated and non-treated systems in fuel cells and water electrolysis tests.

Response: Thank you for your suggestions. The fuel cell performance and durability before crosslinking have been tested as shown in **Figures R5** and **R6** (Figures S19 and S21 in the revised Supporting Information). The water electrolysis performance and durability also have been measured as displayed in **Figures R7** and **R8** (Figures S22 and S23 in the revised Supporting Information).

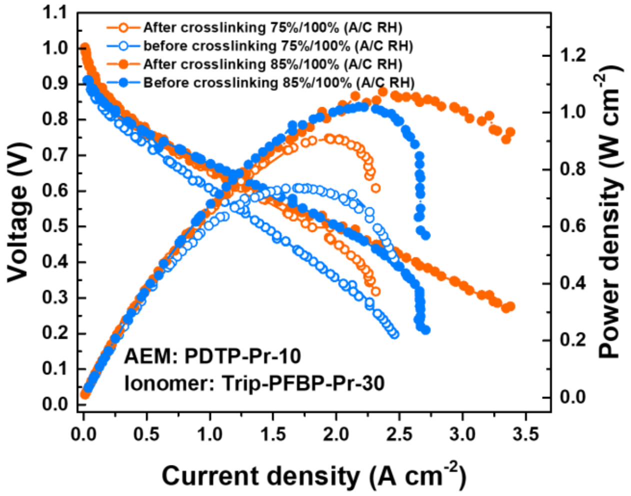

Figure R5. The polarization curves and power density of the MEA (AEM: PDTP-Pr-10; ionomer: Trip-PFBP-Pr-30) before and after crosslinking. Test conditions: cell temperature of 80°C, anode/cathode (A/C) relative humidity (RH) of 75%/100% or 85%/100%, A/C flowrate of H_2/O_2 1000/1000 mL min^{-1}, anode catalyst loading amount of 0.39 mgPtRu cm^{-2}, cathode catalyst loading amount of 0.26 mgPt cm^{-2}.

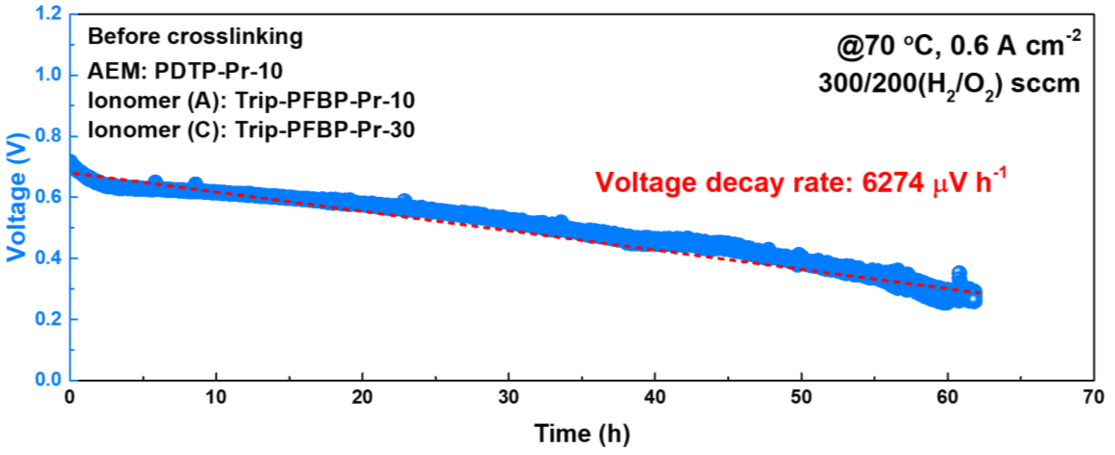

Figure R6. The in-situ durability test of PDTP-Pr-10 AEM (before crosslinking)-based AEMFC at 0.6 A cm^{-2} with Trip-PFBP-Pr-10 (before crosslinking) as anode ionomer and Trip-PFBP-Pr-30 (before crosslinking) as cathode ionomer. Test conditions: cell temperature of 70°C, A/C RH of 94/100%, A/C flowrate of H_2/O_2 300/200 mL min^{-1}, anode catalyst loading of 0.4 mg$_{Pt}$ cm^{-2}, cathode catalyst loading of 0.4 mg$_{Pt}$ cm^{-2}.

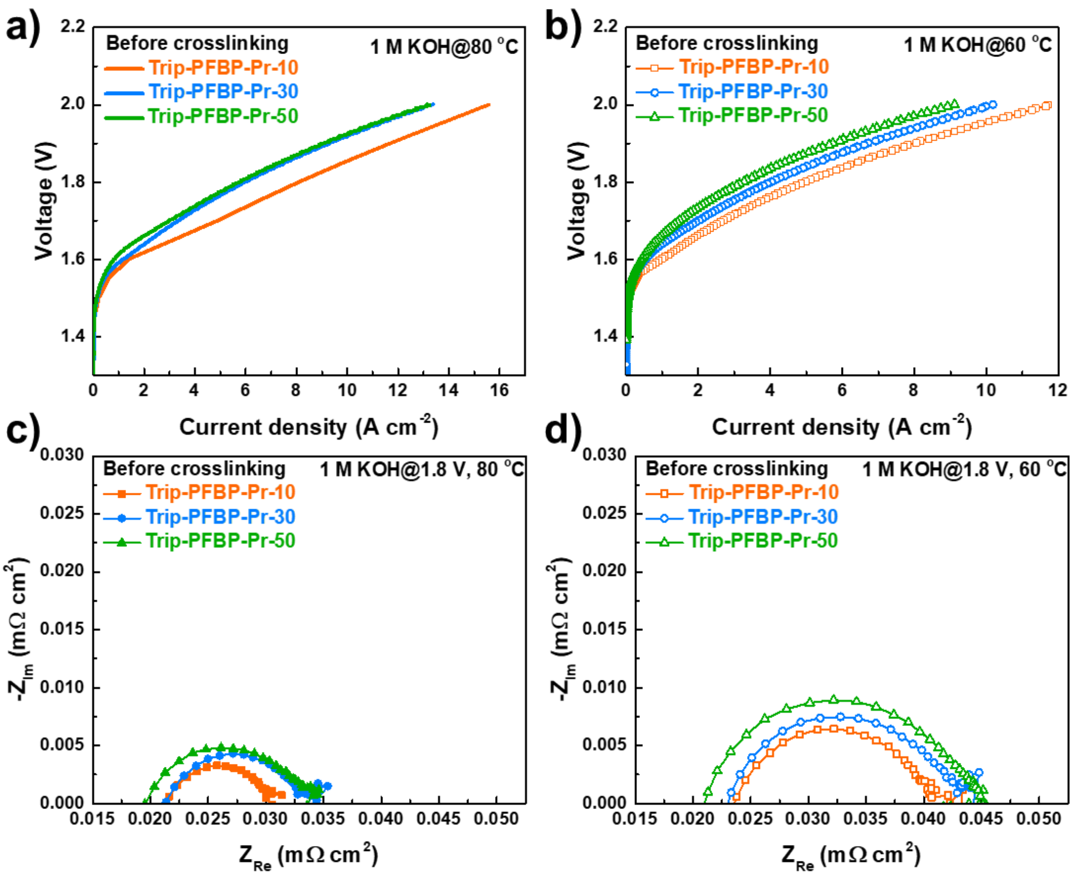

Figure R7. Linear scan voltammograms (LSV) of PDTP-Pr-10 (before crosslinking) AEM (20 μm)-based AEMWE with different ionomers at a) 80°C and b) 60°C in 1 M KOH solution. Potentiostatic electrochemical impedance spectroscopy (PEIS) of PDTP-Pr-10 (before crosslinking) AEM (20 μm)-based AEMWE with different ionomers at c) 1.8 V, 80°C and d) 1.8 V, 60°C in 1 M KOH solution.

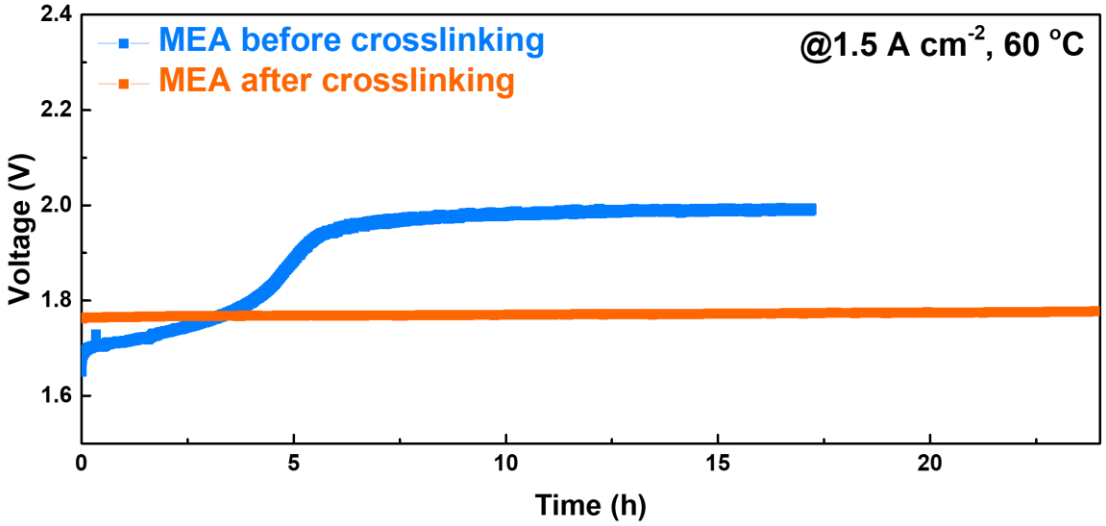

Figure R8. The in-situ durability of x-PDTP-Pr-10 AEM (after crosslinking, 25 μm) and PDTP-Pr-10 AEM (before crosslinking, 25 μm)-based AEMWEs under 60°C at 1 M KOH solution and a current density of 1.5 A cm^{-2}. Test conditions: alkali flow rate of 36 mL min^{-1}, anode catalyst of IrO_2 (2.0 mg cm^{-2}), cathode catalyst of PtRu/C (0.7 mg cm^{-2}).

We added the following statement in page 14 lines 256-260.

"Figure S19 compares the power densities of the MEAs (AEM: PDTP-Pr-10; ionomer: Trip-PFBP-Pr-30) before and after crosslinking at 85%/100% RH and 75%/100% RH. Compared with the non-crosslinked MEA, the MEA after crosslinking possesses a higher power density (1.503 W cm^{-2} vs. 1.019 W cm^{-2} at 85%/100% RH; 0.909 W cm^{-2} vs. 0.735 W cm^{-2} at 75%/100% RH). The improved performance is thought due to the enhanced interaction between the catalyst layer and membrane."

We added the following statement in page 15 lines 289-291.

"*Conversely, the non-crosslinked MEA at the same conditions can be operated for only 60 h with a high voltage decay rate of 6274 μv h^{-1} as shown in Figure S21."*

In page 17, lines 309-320.

"The PDTP-Pr-10 AEM (20 μm)-based AEMWEs with different ionomers (before crosslinking) were used as reference (see Figure S22). Generally, the non-crosslinked MEAs obtain a higher current density over the crosslinked MEAs (Figure S22a, Figure S22b, and Figure 5b). The promoted current density is associated with their low Ohmic resistance (Figure S22c and Figure S22d). Specifically, the non-crosslinked MEA (AEM: PDTP-Pr-10; ionomer: Trip-PFBP-Pr-10) possesses the highest current density of 15.5 A cm^{-2}@2 V, 80°C in a 1 M KOH solution. After thermal treatment, the current density of the MEA (AEM: x-PDTP-Pr-10; ionomer: x-Trip-PFBP-Pr-10) was slightly decreased to 14.34 A cm^{-2} at the same conditions along with an increased R_{ohm} of 22.58 mΩ cm^{-2} and a R_{charge} of 12.28 mΩ cm^{-2}. Due to the dried cathode strategy, x-Trip-PFBP-Pr-30 ionomer-based AEMWE shows a lower current density (11.9 A cm^{-2}@2.0 V) compared with x-Trip-PFBP-Pr-10 ionomer-based AEMWE which is thought to be the low water absorption capability of the cathode ionomer."

Reviewer #2

In this paper, a facile approach to enhance the stability of the catalyst layer by increasing the interactions between the catalyst layer and AEM is proposed. Alkyne groups are introduced in AEM and ionomer, which could form cross-linking by heating. Proper cross-linking would improve the dimensional stability of AEM and the connection between AEM and catalyst layer leading to excellent operational stability in AEMFC and AEMWE. However, there are some questions that should be solved before published.

Response: Thank you for your positive comments. We made point-to-point responses to Reviewer's comments.

1. The text writes that "As heat treatment time increased, the strength of the alkyne absorption peak decreased, suggesting consumption of the propargyl group during the heating process." But triple-bonds are remained in mechanism diagram along with the consumption of alkyne. (Fig.1, Fig.S1) In other words, the crosslinking was done in the wrong way.

Response: Thank you. Theoretically, the thermal crosslinking reaction has two pathways as documented by other reports (*Macromol. Chem. Phys.2015, 216, 2080−2085; Ind. Eng. Chem. Res. 2018, 57, 12511−12518*). One implies a simple trimerization reaction among three acetylene end groups to form an aromatic crosslinker. The other implies a complex coupling reaction to produce a linear crosslinker (*Ind. Eng. Chem. Res. 2018, 57, 12511−12518*). In this work, the adsorption peak at wave number 2120 cm^{-1} decreased after crosslinking suggesting that the main pathway is to form an aromatic crosslinker. To avoid any misunderstanding, we revised Figure 1 and Figure S1.

2. What's the difference between changing the acetylene grafting ratio and crosslinking time in MEA preparation? Which is better?

Response: Thank you. Both acetylene grafting degree and crosslinking time can affect the properties of the polymers and the performance of the MEAs. From our experiences, the polymer with sufficient crosslinking time and a low grafting degree is better. First, the acetylene is hydrophobic and is directly attached to the piperidinium group in Trip-PAP-Pr-m moieties. In this case, the hydrophilicity of piperidinium decreased and therefore high grafting degree is not beneficial for the ion transport. Additionally, the grafting onto the piperidinium will inevitably destroy the conformation of the piperidinium resulting in decreased stability. In conclusion, low grafting with high crosslinking time is better.

3. Whether cross-linking between ionomers would increase the mass transfer resistance? And what's the evidence?

Response: Thank you. In this work, we did not explore the effect of crosslinked ionomer on mass transfer resistance. However, Feng Yan's group reported a work (*J. Mater. Chem. A, 2022, 10, 13355–13367*) that claimed that the rigid anthracene-based ionomer after crosslinking has a higher

free volume which is beneficial for the gas permeability resulting in a low mass transfer resistance. However, if the ionomer has a dense structure, the mass transfer resistance may increase. It is a good point to analyze the effect of crosslinking structure on the mass transfer resistance. However, the main purpose of this work is to promote the stability of the catalyst layer for a long operational life. In the future, we will study this research as you mentioned.

4. “Meanwhile, the weak connection between the catalyst layer and AEM results in detachment of the catalyst layer, as displayed in the inset pictures in Figure 1a. To evaluate the interactions with the catalyst layer, the peeling strength of the catalyst layer was measured using a peel-off test.” “The higher peeling strength of MEA suggests stronger interactions between catalyst layers”. What is the thickness of the catalyst layer tested? Whether the test is between catalyst layers or between catalyst layers and AEM? If between catalyst layers, why do the interactions between the same ionomers change as the substrate AEM changes in Fig. 2c?

Response: Thank you. The thickness of the catalyst layer has been confirmed to be about 6.6 μm using SEM as displayed in **Figure R9** (Figure S12 in the revised Supporting Information). The peeling-off measurement is a comprehensive test including the interaction between catalyst layers and between the catalyst layer and AEM. We cannot separate these two interactions and study them individually. If the interaction between membrane and catalyst is strong, the peeling-off measurement is for the interaction between catalyst layers. If the interaction between the catalyst layer is strong, the peeling-off measurement is for the interaction between the membrane and the catalyst layer. However, the forces for these two interactions are not that big. Therefore, it will affect each other.

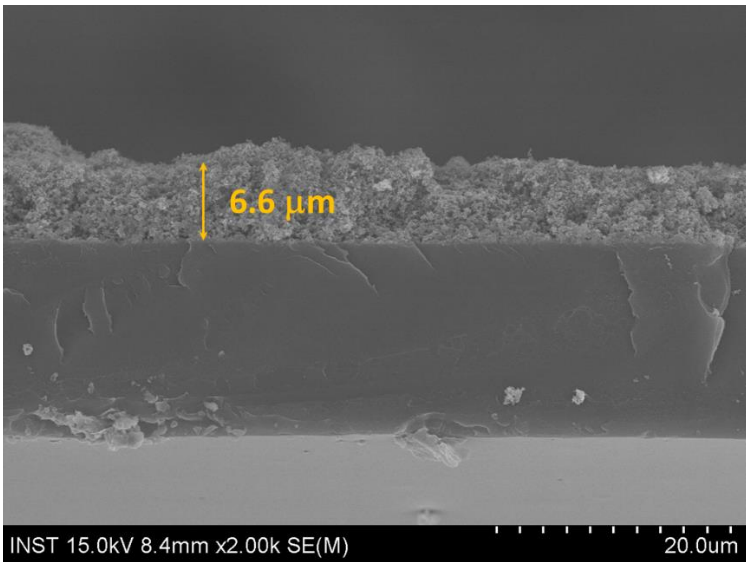

Figure R9. The cross-sectional image of the MEA for the peeling-off measurement.

5. “The oxygen bubbles generated in the anode will crash the catalyst causing the dispersion or detachment of the catalyst due to poor connection of catalyst layers, especially at a high current density (see Figure 5a). Therefore, stabilizing the catalyst layer is crucial for the long-term operation of AEMWEs.” There is still poor connection in anode used in AEMWE.

Response: Thank you. On the anode side, we used x-PFBP-Pr-30 as ionomers and x-PDTP-Pr-10 as AEM. Despite the interaction between the x-PFBP-Pr-30 ionomer and x-PDTP-Pr-10 membrane is not as strong as the x-PDTP-Pr-50 membrane and x-PFBP-Pr-30, 50 ionomers, the stability of the catalyst layer is much higher than the non-crosslinked ionomers and membranes. Otherwise, the AEMWE cannot be operated at a high current density for over 1000 h. In Figure 5a, we would like to emphasize the importance of the stability of the anode catalyst layer because it is easily destroyed by the generated oxygen bubbles. To avoid the misunderstanding, we redrew this diagram in Fig. 5a in the revised manuscript.

6. There are still syntax errors that need to be corrected.

Response: Thank you. We have carefully checked the syntax errors in the manuscript.

oc-2023-014909.R2

Name: Peer Review Information for "Stabilizing the catalyst layer for durable and high performance alkaline membrane fuel cells and water electrolyzers"

Second Round of Reviewer Comments

Reviewer: 2

Comments to the Author

The authors have made efforts and revised the manuscript, the revised manuscript is suitable for publication.

Reviewer: 1

Comments to the Author

The publication of this work is recommended.

Author's Response to Peer Review Comments:

Dear Professor Editor

Thank you for your fast decision on our revision. We have changed the format issues of our revised manuscript according to your guideline and attach the new manuscript file for your final decision. Thank you again and if you have any further queries, please let me know. Thank you.

Best regards,

Young Moo Lee
